# β‑BaCO_3_ Facilitates a Topochemical Reaction Route for the High-Temperature Synthesis of Perovskite-Type (Ba,Ca)(Zr,Ti)O_3_ via Oxycarbonate Intermediates

**DOI:** 10.1021/acs.chemmater.5c03257

**Published:** 2026-04-02

**Authors:** Anna M. Paulik, Marc Widenmeyer, Sylvia L. Kunz, Magdalena O. Cichocka, Ute Kolb, Jurij Koruza, Oliver Clemens

**Affiliations:** † Institute for Chemistry and Technology of Materials, 27253 Graz University of Technology, Stremayrgasse 9, 8010 Graz, Austria; ‡ 26536 Technical University of Darmstadt, Institute for Materials Science, Materials and Resources, Peter-Grünberg-Straße 2, 64287 Darmstadt, Germany; § University of Stuttgart, Institute for Materials Science, Materials Synthesis Group, Heisenbergstraße 3, 70569 Stuttgart, Germany; ∥ Technical University of Darmstadt, Institute of Applied Geosciences, Electron Crystallography Group, Schnittspahnstraße 9, 64287 Darmstadt, Germany

## Abstract

Ferroelectric oxides from the perovskite-type (Ba,Ca)­(Zr,Ti)­O_3_ system are emerging as one of the upcoming lead-free piezoelectrics for future sensing and actuating applications. Improving their functionality requires a precise adjustment of precursor choice, pretreatment, as well as reaction and sintering times and temperatures to optimize the defect chemistry and microstructure of the material. In this respect, understanding the reaction pathways of the precursors has a crucial impact on the final functional properties. In this article, we show that the high-temperature modification of the BaCO_3_ precursor can strongly influence the reaction by facilitating a topochemical reaction pathway via the formation of an intermediate oxycarbonate phase of BaTiO_3–*x*_(CO_3_)_*x*_ (*x* ≈ 1/3). This results from the diffusion of Ti^4+^ and 2 O^2–^ into the material in combination with CO_2_ outgassing, distinguishing this reaction path from the previously reported diffusion mechanism via an intermediate phase of Ba_2_TiO_4_, which happens in parallel. We used high-temperature in situ X-ray diffraction and automated diffraction tomography to derive a structural model of this previously unknown intermediate oxycarbonate phase, which can be understood as a trigonal distortion variant of the cubic perovskite structure, where the trigonal distortion originates from the planar nature of the carbonate anion. This mechanism might have implications on how the BaCO_3_ precursor and its decomposition behavior could decisively influence particle morphology and composition obtained prior to sintering of (Ba,Ca)­(Zr,Ti)­O_3_ ferroelectric oxides.

## Graphical abstract

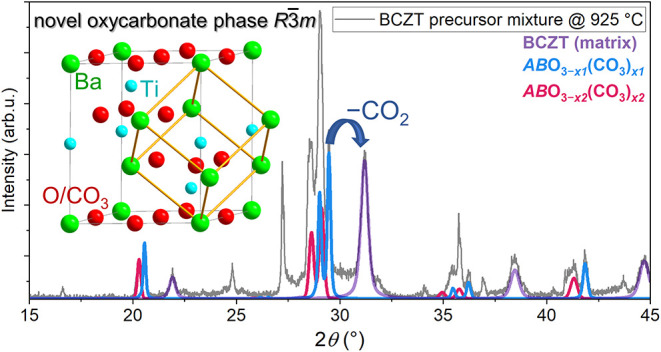

## 1 Introduction

Perovskite materials are currently one of the most widely investigated classes of materials, due to their capability to tune the functional properties by adjusting their structure and composition in wide ranges.^1^ This exceptional versatility led to promising developments in a variety of different fields. This includes solar cell technologies,^2,3^ photocatalysis,^4,5^ and electrolytes for all-solid-state batteries.^6^ Inorganic perovskite oxides are also the most widely used materials for capacitor dielectrics, as well as piezoelectric transducers, actuators, and sensors.^1,7^ Though Pb­(Zr,Ti)­O_3_ (PZT) exhibits particularly attractive functional properties and is the state-of-the-art material for most industrial piezoelectric applications, the dangers of lead, posed to human health and the environment, require investigation and finding of lead-free alternatives. Even though critical obstacles persist in the development of lead-free ferroelectric materials, especially regarding the reproducibility and stability of the mechanical and functional properties, significant progress has been made in the past few decades, which inspired a transition into application-based research.^8^ Lead-free products are being developed for critical areas like ultrasonic imaging, actuating, and sensing, while new technologies like piezoelectric energy harvesting are also being established.^9,10^ Among the possible alternatives to PZT, BaTiO_3_-based systems represent one of the most promising candidates and have been chemically modified to improve the functional properties.^11,12^

(Ba,Ca)­(Zr,Ti)­O_3_ (BCZT) is an emerging perovskite oxide with certain ferroelectric properties that surpass those of the lead-based systems. The material can be structurally understood as BaTiO_3_ with Ca^2+^ and Zr^4+^ partially isovalently substituting Ba^2+^ and Ti^4+^ on the *A*- and *B*-sites, respectively. This particular modification of BaTiO_3_ was first investigated in 1954 ^13^ and has been rediscovered as a possible candidate for ferroelectric applications in 2009, when outstanding piezoelectric properties were first published for compositions close to the polymorphic phase boundaries.^14^

Due to the strong dependence of their functional properties on defect chemistry, structural parameters, and microstructure, the synthesis conditions of perovskite oxides play a vital role and have to be optimized to obtain high-performance materials without elemental losses and/or the inclusion of contaminations.^8^ This is of particular importance for implementing advanced doping strategies using Fermi level engineering.^15^

Synthesis of perovskite oxides can be achieved through different methods, including aqueous methods,^16–18^ sol–gel synthesis,^19–21^ oxalate coprecipitation,^22^ synthesis from polymeric organic precursors,^23^ and through solid-state reaction from oxide and carbonate precursors.^24,25^ Due to the complexity of the precursors used and the introduction of carbon sources into the synthesis process, the reaction can comprise the formation of intermediate phases, among them oxycarbonates. A variety of different oxycarbonate phases, like BaO_1–*x*_(CO_3_)_*x*_,^17^ BaTiO_3–*x*_(CO_3_)_*x*_,^26^ and BaTiO_2.5_(CO_3_)_0.5_,^27–30^ have been identified for various reactions which apply organic solvents or organic precursors. These phases are typically only stable up to about 600 °C; remarkably, none of them have been observed in samples manufactured via a solid-state reaction route using inorganic carbonates (BaCO_3_, CaCO_3_) as precursors, which is the most commonly used process to synthesize bulk BaTiO_3_-based ceramics and demands temperatures above 1000 °C.^24,31,32^

The solid-state reaction route, including the sequence of intermediate phase formation, is accessible through high-temperature in situ X-ray diffraction, and this method has been applied to successfully identify the formation reactions and transient phases of several different inorganic ceramic systems.^31,33–36^ In their study on the lead-free piezoelectric (K,Na)­NbO_3_ system, Thong et al. used high-temperature in situ X-ray diffraction to identify competing solid-state reactions between reactants as a likely origin for the poor homogeneity and lack of reproducibility of the ferroelectric performance observed in this system. During the solid-state reaction process of BaTiO_3_-based materials, nonperovskite intermediate phases like Ba_2_TiO_4_ are known to form,^32,37^ and in multicomponent systems like BCZT, perovskite phases of deviating composition are also present.^31,38^ An alternative reaction route via the formation of oxycarbonates has not been considered to date due to the aforementioned reasons. Only recently, we reported a first indication that such phases might also play a role in reaction sequences starting from inorganic precursors.^31^

In this article, we describe the formerly unknown intermediate oxycarbonate phase of *AB*O_3–*x*_(CO_3_)_*x*_, which is formed during the solid-state synthesis of BCZT, in full detail. We show that this phase fundamentally differs from the oxycarbonate phases which have previously been described, not only in its structure but also in its temperature stability, which exceeds 1000 °C. We investigated the formation of this phase using high-temperature in situ X-ray diffraction, characterized its structure by Rietveld refinements and a three-dimensional electron diffraction method (3D ED), and described the topochemical reaction process leading to its formation and subsequent decomposition. Quenching experiments allowed us to confirm the presence of trigonal distorted oxycarbonates at ambient temperature, showing an even higher complexity than observed during in situ studies at high temperature. Our findings show that a topochemical reaction via intermediate oxycarbonate phases is in principle possible for BaTiO_3_-based materials, which challenges our fundamental understanding of the BaTiO_3_ formation reaction process.

## 2 Experimental Section

### 2.1 Synthesis

Homogenous mixtures of high-purity precursors, BaCO_3_ (99.95%, Alfa Aesar), CaCO_3_ (99.95%, Thermo Scientific), ZrO_2_ (99.978%, Thermo Scientific), and TiO_2_ (99.5%, rutile, Alfa Aesar), were prepared with the Ba_0.82_Ca_0.18_Zr_0.08_Ti_0.92_O_3_ stoichiometry (BCZT; also marked as BZT-60BCT in the literature). Granulometric measurements of all precursors were performed before mixing, using dynamic light scattering (Cilas 1064). The precursors CaCO_3_ and ZrO_2_ were individually premilled before use in ethanol for 4 h in a planetary mill (Retsch PM400) at 175 rpm using 3 mm yttria-stabilized zirconia (YSZ) milling balls. The measured *d*_*50*_ values of the precursor particle sizes were 1.24 μm (BaCO_3_), 1.27 μm (CaCO_3_), 0.89 μm (ZrO_2_), and 0.93 μm (TiO_2_). More details on the precursor preparation can be found in our previous publication.^31^

The precursor powders were dried overnight at 400 °C before being weighed stoichiometrically. The powder mixture, which was used for in situ XRD measurements, was prepared by planetary ball milling stoichiometric precursor mixtures in ethanol for 4 h at 200 rpm using 3 mm YSZ balls, resulting in a *d*_*50*_ of 0.70 μm. For ex situ quenching experiments, a powder mixture of the same composition was homogenized on a rolling bench (GERMATEC) for 24 h using 10 mm YSZ balls, which yielded a *d*_*50*_ particle size of 0.96 μm.

For quenching, a loosely packed sample of approximately 1 g was heated in a tube furnace (Carbolite Gero) on an alumina sample holder lined with platinum foil (uncovered). The temperature program was set to follow the one used for the in situ XRD measurement (see next section), skipping the dwell time at 300 °C during heating. The sample was then air-quenched at the end of the 61 min dwell period at 925 °C. Micro-Raman spectra of quenched samples were collected on a LabRAM Horiba HR Raman spectroscope HR800 (Horiba Jobin Yvon GmbH) using a green laser at 514.5 nm.

### 2.2 X-ray Diffraction

In situ XRD measurements were performed on an XRDynamic 500 diffractometer equipped with a HTK 1500 heating cell, using a Cu *K*_α_ radiation and a Pixos 2000 detector (Anton Paar), equipped with a Ni filter for *K*_ß_ removal. Diffractograms were recorded with a step size of 0.01° over a 2θ range from 15° to 70° with a scanning speed of 0.9°/min, resulting in a measurement time of 61 min. The sample was heated at a rate of 5 °C/min between measurement and held for the duration of each measurement at the respective temperature. Measurements were performed at room temperature (before and after applying the temperature program), at 300 °C, 600 °C, and from 800 to 1100 °C in steps of 25 °C. Up to 1400 °C, diffractograms were recorded in steps of 50 °C.

Room-temperature X-ray diffractograms of the quenched sample were collected using the Miniflex 600 diffractometer (Cu *K*_α_, equipped with a Ni filter for *K*_ß_ removal, Rigaku) with a scanning speed of 5°/min and a step size of 0.01°.

Ambient-temperature high-resolution synchrotron diffraction data were recorded on the sample quenched from 925 °C to room temperature using the ID22 beam line of the European Synchrotron Radiation Facility (ESRF, Grenoble, France) with a wavelength of λ = 0.35430(6) Å and a binning interval of 0.001°.^39,40^

The software Match! (Crystal Impact), as well as the Crystallography Open Database (COD) and the Inorganic Crystal Structure Database (ICSD) were used for identifying the phases. This phase analysis served as the starting point for the subsequent Rietveld analysis, which was performed using the software TOPAS 6 (Bruker), using a modified fundamental parameters approach with the instrumental broadening learned from a scan of a reference sample.^41,42^ Sample-related broadening was modeled by using Voigt functions with cos^–1^(θ) and tan­(θ) angular dependence of broadening to adjust for crystallite size and microstrain effects on the reflection profile, respectively. For phases with crystal structures reported before, the structural data were taken from the ICSD, while only adjusting lattice parameters; for the trigonal perovskite-related phases with composition of approximately BaTiO_3–*x*_(CO_3_)_*x*_, the structural models were derived as will be described in the article. The details of the analysis of the synchrotron diffraction data are reported in the respective section.

In general, we would like to make the reader aware that the errors given throughout the manuscript, e.g., for the diffraction analyses, are numerical errors for the refinement routine and do not necessarily present a confidence interval. Such intervals are hard to predict realistically for the current study, since small changes in the experimental protocol (heating and cooling rates, measurement times, quenching speed) will likely influence the detailed carbonate content and phase homogeneity, and by this, lattice parameters as well as “apparent reflection widths” of multiphase oxycarbonates on quenching.

### 2.3 Three-Dimensional Electron Diffraction

Three-dimensional electron diffraction (3D ED) and energy-dispersive X-ray spectroscopy (EDX) were carried out using a Tecnai F30 ST transmission electron microscope (TEM) with an acceleration voltage of 300 kV, corresponding to an electron wavelength of 0.0197 Å. The microscope was operated in μ-STEM mode with a 200 nm beam diameter, using a gun lens setting of 8, spot size 6, and a 10 mm C2 condenser aperture. Annular dark-field (ADF) imaging with a Fischione detector was used to visualize crystal tracking.

Electron diffraction data were acquired using electron beam precession at a semiangle of 1° and a frequency of 100 Hz, controlled by the Nanomegas Digistar system. A cryo transfer holder from Simple Origin, with a tilt range of ± 50°, enabled the collection of 3D ED data. ED patterns were recorded on a Gatan US4000 CCD camera with 4k × 4k resolution. The PyFast-ADT module^43^ was used to control the data acquisition process.

EDX spectra were collected using an EDAX Si (Li) detector at a stage tilt of 20°. TEM samples were prepared by dispersing the material in ethanol and depositing it onto a copper grid coated with a continuous carbon film and subsequently cooled in liquid nitrogen.

Reciprocal space reconstruction and reflection file generation were performed using PETS2.^44^ The initial structure solution was achieved with SIR2014,^45^ followed by refinements using ShelXle,^46^ a graphical interface for SHELXL.^47^ The final structure model was refined by using the kinematical approximation.

## 3 Results and Discussion

### 3.1 Identification and Characterization of Oxycarbonate Phases from High-Temperature In Situ X-ray Diffraction Experiments

To help understand the details of the genesis of the oxycarbonate phases, identified in this work, we start by summarizing our previous findings on the phase evolution of BCZT with the composition Ba_0.82_Ca_0.18_Zr_0.08_Ti_0.92_O_3_ via in situ powder diffraction. In our previous study,^31^ we found that the solid-state formation reaction of BCZT from BaCO_3_, CaCO_3_, TiO_2_, and ZrO_2_ precursors starts at approximately 600 °C with the formation of CaZrO_3_, CaTiO_3_, CaO, and a small amount of CaZrTi_2_O_7_ intermediate phase, which is consistent with previous literature on the Ca­(Zr,Ti)­O_3_ system.^31,48,49^ CaZrTi_2_O_7_ and CaTiO_3_ decompose at 700 °C and a small amount of a BaZrO_3_-rich perovskite is formed above 800 °C. Due to the low amounts of Ca and Zr in the system (18% on the *A*-site and 8% on the *B*-site, respectively) and the large fraction of Ca- and Zr-containing intermediate phases, already present at this temperature, the remaining unreacted precursors consist predominantly of BaCO_3_ and TiO_2_. Once the transition temperature of *α*-BaCO_3_ to trigonal *R*3̅*m*-type *β*-BaCO_3_ at approximately 900 °C is reached,^50^ a partial change in reaction mechanisms occurs. The process deviates from the previously described reaction route of BaTiO_3_,^32^ and additional prominent reflections are indicated in the pattern. These reflections could neither be assigned to known phases, contained within the sample, nor matched with any phase from structure databases (Figure 1).

**Figure 1:**
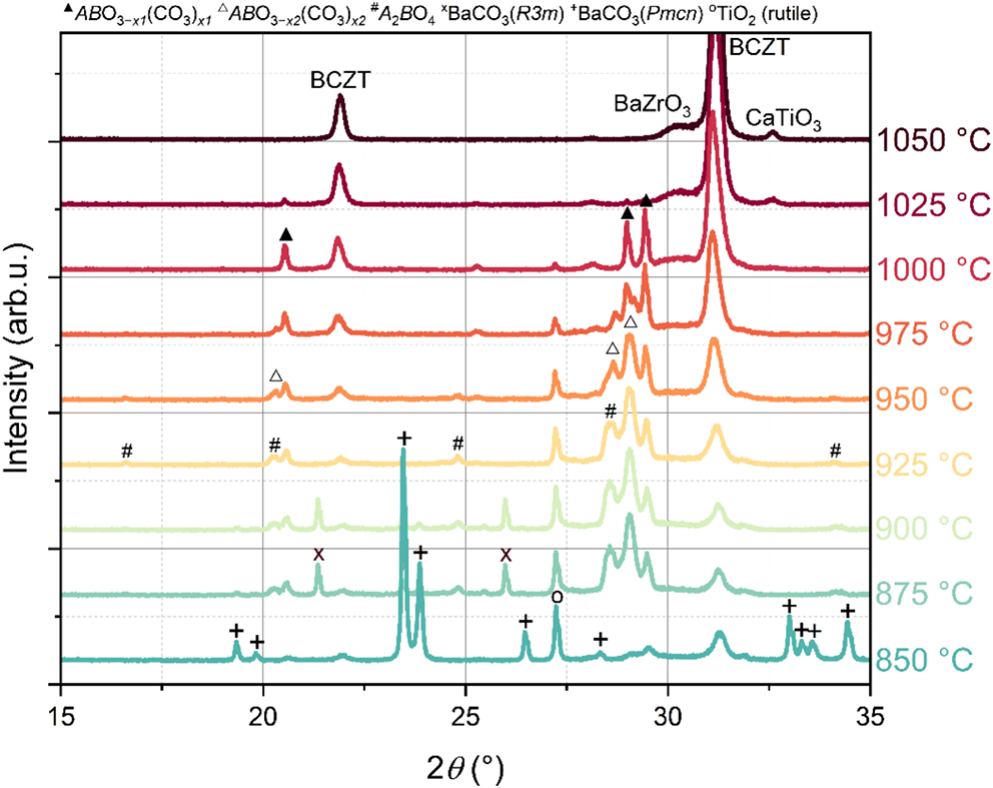
In situ X-ray diffractograms (Cu *K*_α_) of a homogenized BCZT precursor mixture in the range of oxycarbonate formation and decomposition. Different aspects of this data were also discussed in our previous publication.^31^

The formation of these unknown phases is indicated by the appearance of intense main reflections in the 2θ angular range between 27.5° and 30° beyond 900 °C, in addition to further reflections over the whole angular range. We emphasize that only a small fraction of the intensity of the main reflections can be explained by the formation of the ortho-titanate Ba_2_TiO_4_, which is a nonperovskite-type phase that is present in our sample in a small amount, but does not match the additional reflections observed (see Figure S1). On increasing the temperature to 1000 °C, the diffraction pattern only contains sharp reflections belonging to these so far unknown phases and perovskite-type compounds (apart from a small amount of rutile-type TiO_2_, Figure 1), and thus allows for a closer structural analysis of the observed phases. For this, we performed an indexing^51^ starting from determining the reflection positions by performing a fitting of these sharp reflections. This indexing revealed that the underlying unit cell can be described with a *R*-centered trigonal cell with space group *R*3̅*m* (or alternative space groups with identical extinction conditions) and lattice parameters of *a* = 6.0595(2) Å, *c* = 7.5915(3) Å (*c/a* = 1.253). Since we started with an overall sample composition of *A*_1_ *B*_1_ *X*_3_ (*A* = Ca^2+^, Ba^2+^; *B* = Ti^4+^, Zr^4+^; *X* = O^2–^, CO_3_^2–^), and the additional phases found are perovskite-type phases with a composition of *A*_1_ *B*_1_O_3_, simple stoichiometric triangulation leads to the conclusion that (in the absence of amorphous phases or crystalline phases with composition *A*_*n±m*_ *B*_*n*_O_3*n*±*m*_, (*m, n* being integer numbers)) this phase must also contain *A*- and *B*-site cations in a ratio of approximately 1:1. Already a visual inspection of the pattern reveals that in comparison to the perovskite main phase, the observed trigonal phase has similar reflection groups as a perovskite-type phase, which appear shifted to lower angles; further, the intensity pattern obtained by summing the intensities of the split intensities within a reflection group is similar to the intensity pattern of BaTiO_3_ (see Figure S2). For a cubic BaTiO_3_ perovskite, it is important to note that the intensity of the powder diffraction pattern is dominated by the electron density contrast of Ba to Ti, while the oxygen species only have a lower contribution to the overall intensity pattern (see Figure S3). This gives a strong hint that the observed trigonal phase possesses a similar positional pattern of Ba and Ti as found in perovskite-type materials.

*R*3̅*m* is a maximum *translationengleiche* subgroup of the *Pm*3̅*m* perovskite-type structure. The *c*-axis of the hexagonal setting of the trigonal cell would correspond to the [111] direction of the cubic cell, and the *a*-axis would correspond to the [11̅0] direction accordingly; consequently, a *c/a*-ratio of (3/2)^0.5^ = 1.225 corresponds to a pseudocubic unit cell. The primitive cell of the *R*-centered trigonal cell would have a volume of ∼80.5 Å^3^ per *ABX*_3_ unit (corresponding to a pseudocubic lattice parameter of 4.32 Å), which is considerably larger (by 13.3 Å^3^) than the volume of the cubic perovskite main phase observed at 1000 °C (67.2 Å^3^ corresponding to *a* = 4.06 Å). Since the perovskite structure is structurally derived from a *ccp*-packing of close-packed *AX*_3_ layers,^52,53^ a volume difference of such an extent can only be reasonably explained by a difference in chemical composition of this phase with respect to the nature and orientation of the species forming the close packing. On the other hand, the integral intensities of reflection groups match to what is observed for the perovskite main phase, indicating that the strongest scatterers, i.e., the *A*- and *B*-site cations, form a sublattice within the *R*3̅*m* phase, which must be basically similar to the perovskite phase. Thus, the deviation from a perovskite phase must mainly originate from structural features of the anion sublattice. Indeed, we found that the intensity pattern observed can be already approximately fitted with the structural model as specified in the caption of Figure S2, containing only Ba as a single *A*-site cation and Ti as a single *B*-site cation (on the special sites 3*a* and 3*b*, respectively, without using any anions).

As indicated from the TG/DTA analysis shown in our previous report,^31^ the oxycarbonate intermediate phases are observed within a temperature range, which shows a strong release of CO_2_ from BaCO_3_. These measurements clarify that the formation and disappearance of the oxycarbonate phases is governed by carbonate decomposition, as also indicated from the concomitant mass spectrometry analysis shown in ref 31. Further, the previously unknown *R*3̅*m* perovskite-related phases only appear after BaCO_3_ has transformed to its high-temperature modification (*β*-BaCO_3_ previously reported to crystallize in space group *R*3̅*m* or *R3m* ^54^); it is remarkable that *β*-BaCO_3_ is only observed in a very narrow temperature window, and once it disappears, the perovskite-related trigonal phases are being formed. Due to the similarity of space groups, it is worth inspecting the structural relationships in more detail. On first inspection, the lattice parameters of *β*-BaCO_3_ are significantly different from those of the trigonal phase (*a* = 5.201 Å, *c* = 10.657 Å (*V*_*f.u*._ = 83.17 Å^3^, *c*/*a* = 2.049) reported for *β*-BaCO_3_ at 900 °C^54^ compared to *a* = 6.0595(2) Å, *c* = 7.5915(3) Å (*V*_*f.u*._ = 80.47 Å^3^, *c*/*a* = 1.253) at 1000 °C determined for the trigonal phase). However, the unit cells of both structures contain three formula units of Ba^2+^ ions, and the volumes per formula *ABX*_3_ unit “*V*_*f.u*._” are very similar. The substructure of Ba^2+^ in a trigonal perovskite and in *β*-BaCO_3_ can be understood as an arrangement similar to atoms within a *ccp*-stacked packing with a stacking sequence *ABC* (see Figure 2). Thus, the transformation of trigonal *β*-BaCO_3_ to a cubic BaTiO_3_ requires the release of CO_2_ in combination with the incorporation of TiO_2_ as Ti^4+^ and 2 O^2–^ into the matrix, with an additional relaxation of the lattice parameters to a *c/a* ratio close to the ideal value of (3/2)^0.5^ = 1.225. This can be described schematically by the following reaction equation in Kröger-Vink notation (1), using the Wyckoff sites and carbonate location of a hypothetical perovskite-type *β*-BaCO_3_ (Wyckoff sites that are unoccupied in pure *β*-BaCO_3_ are marked as interstitial, *i*)­

1 $${Ba}_{3a}^{x}+{\left(CO_{3}\right)}_{CO_{3}}^{x}+x{\left(TiO_{2}\right)}_{Surf}\to {Ba}_{3a}^{x}+x{Ti}_{i,3b}^{••••}+\left(1-x\right){\left(CO_{3}\right)}_{CO_{3}}^{x}+3xO_{i,9d}^{\prime \prime }+xv_{CO_{3}}^{•••}+xCO_{2}↑$$

**Figure 2:**
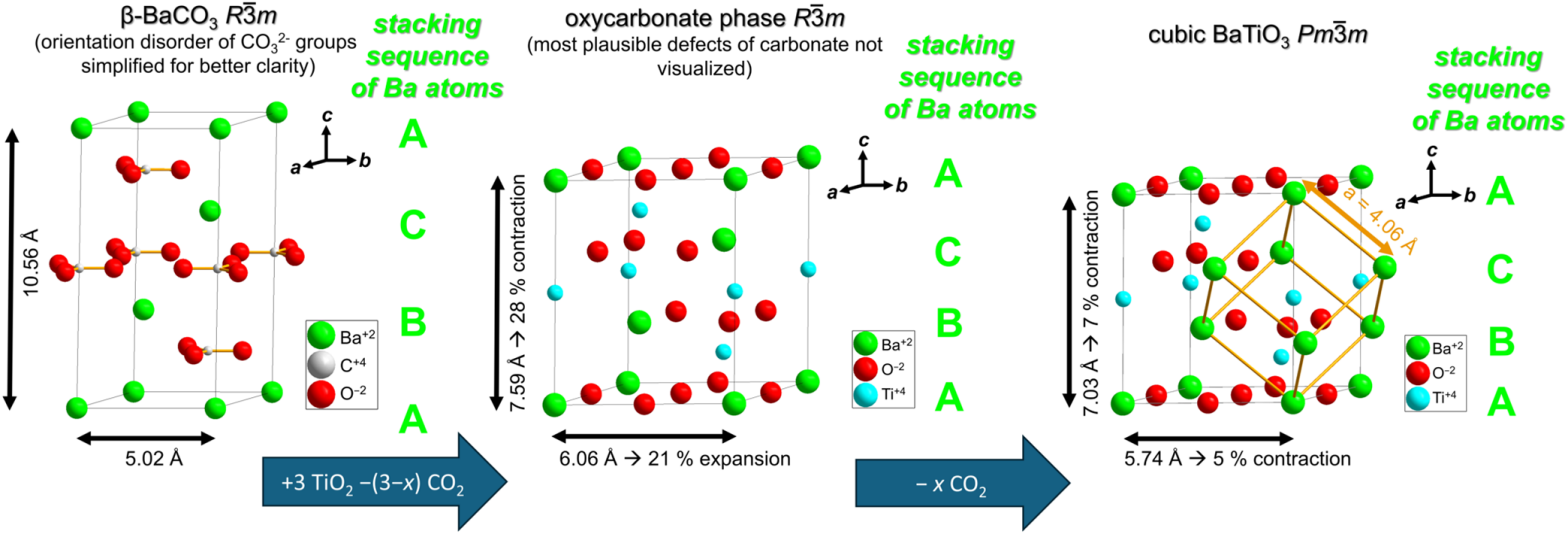
Conceptual visualization of the transformation of the high-temperature modification *β*-BaCO_3_ to BaTiO_3_ via the oxycarbonate BaTiO_3–*x*_(CO_3_)*_x_*, showing the structural relationships between the three phases and contractions/expansions required along the different crystallographic axes at 1000 °C.

The overall reaction scheme is visualized in Figure 2. As indicated from the reaction eq 1 above, the formation of such an intermediate phase would be of a topochemical nature, since it only requires a partial reorganization and interdiffusion within an already existing lattice. This is also indicated from the reflection widths of the high-temperature phase of *β*-BaCO_3_ in comparison to the trigonal oxycarbonate phase, which indicate a highly crystalline nature of this phase right after formation; the reflection widths of *β*-BaCO_3_ at 850 °C and of the oxycarbonate phase at 1000 °C are similar. Further, the width of the oxycarbonate phase reflections seems to be dominated by crystallite size broadening and not by strain broadening. However, the angular dependence of broadening (distinguishing between microstrain and crystallite size) should not be overinterpreted due to the necessity to limit the angular range of the measurement. In contrast, perovskite phases formed from solid-state reaction prior to the “*β*-BaCO_3_ reaction route” show significantly broader reflections, with lower crystallite sizes/higher microstrain. Considering kinetical aspects, one can further assume that by the release of CO_2_, the mobility of Ti^4+^ and O^2–^ within *β*-BaCO_3_ would be enhanced, thus facilitating the phase formation of the oxycarbonate phase.

The observation of a trigonal (thus symmetry-lowered) perovskite at a temperature of 900–1000 °C is untypical, since most perovskite-type materials with distortions which can be derived from the cubic aristotype structure^55^ tend to adopt the high symmetry scenario on temperature increase, e.g., the phase transitions for tetragonal BaTiO_3_ to the cubic modification beyond 120 °C.^56^ However, the incorporation of significant amounts of nonspherical planar carbonate ions could plausibly explain such a trigonal distortion: an orientation of the plane of C–O bonding perpendicular to the *c*-axis containing the 3-fold symmetry in a perovskite-related structure would provide a plausible origin for the symmetry lowering of the system. Furthermore, one must consider that CO_3_^2–^ groups within the oxycarbonate would require more space than an isovalent O^2–^ ion and would orient perpendicular to the *c*-axis of the trigonal cell. The increase in cell size along the trigonal *a*-axis in comparison to a pseudocubic trigonal perovskite unit cell (see Figure 2) strongly supports the argument of symmetry lowering as an origin from the orientation of the carbonate species within this phase.

Taking the above considerations into account (together with the fact that we could not perfectly stabilize the oxycarbonate phase by quenching to ambient temperature, see Section 3.2), we used a structural model with disorder of weak scatterers within the BaO_3–*x*_(CO_3_)_*x*_ *AX*_3_-layer together with a *B*_iso_ parameter constraining the thermal motion of the atoms of this phase, which gives a reasonable fit of the intensity observed for the pattern (see Figure 3 and Table 1). This indicates that the degree of anion disordering of oxide and carbonate species is larger and thus also leads to a fairly large displacement of the heavy scatterers Ba and Ti from their ideal positions; further, we emphasize that including partial substitution of Ba by Ca or Ti by Zr did not improve the fit any further, and converged to basically full occupancy of the *A-* and *B*-sites by Ba and Ti. Thus, we assume that the oxycarbonate phase contains mainly Ba and Ti as the respective *A*- and *B*-site cations.

**Figure 3:**
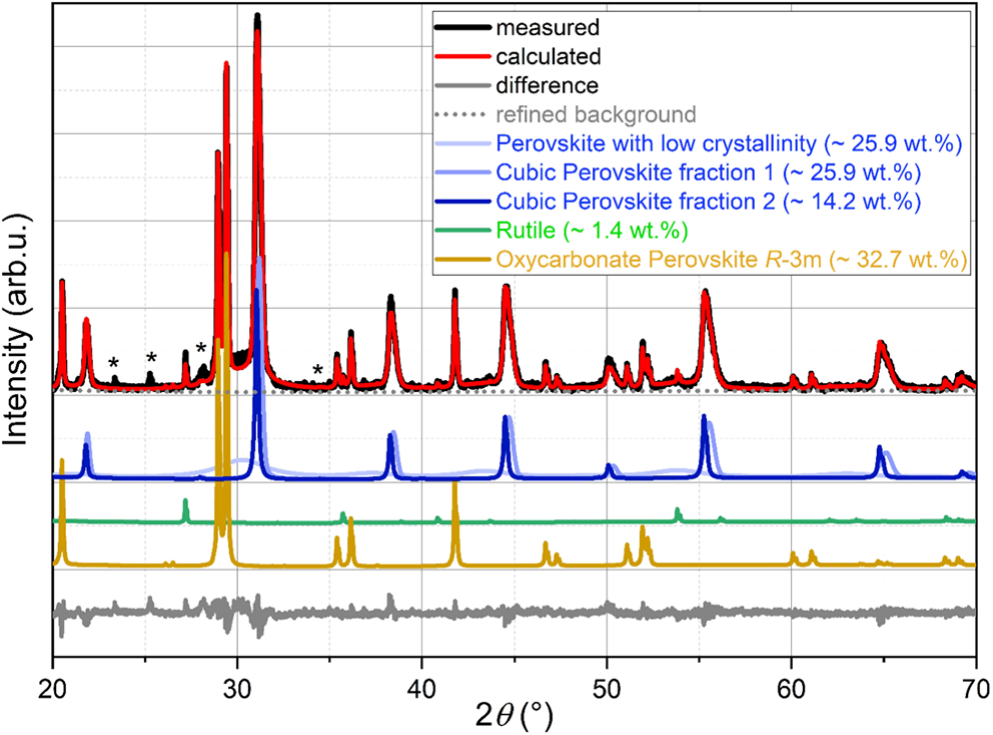
Fit of the diffraction pattern of BCZT at 1000 °C (Cu *K*_α_) together with the structural model of the oxycarbonate phase; the green fit curve belongs to the partial fit curve of this phase (*R*_Bragg_ = 9%), *unknown/unidentified reflections (only the most intense are indicated).

**Table 1:** Atomic Positions in the Trigonal Oxycarbonate Perovskite, Identified from the Fit of the Diffraction Pattern of BCZT at 1000 °C, Space group *R*3̅*m*; *a* = 6.0595(2) Å, *c* = 7.5915(3) Å, Shown in Figure 3

atom	species	Wyckoff site	*x*	*y*	*z*	occupancy	*B*_iso_ [Å^2^]
Ba	Ba^2+^	3*a*	0	0	0	1	6.6(1)
Ti	Ti^4+^	3*b*	0	0	1/2	1	
C^b^	C	6*c*	2/3	1/3	0^a^	0.96(2)	
O	O^2–^	9*d*	1/2	0	0	0.65(2)	

a Site 6*c* has a degree of freedom along the *z*-coordinate, which converged close to a value of 1 and was thus fixed for the final refinements.

b This atom species must not necessarily be a carbon atom but could also be a displaced oxygen; it would be a position suitable for a carbon atom in between oxide ions, resembling the scenario in a carbonate ion.

After deriving a structural model of the trigonal oxycarbonate phase from the in situ measurement at 1000 °C, we could also identify a second, most plausibly even carbonate-richer phase in the measurements in the temperature range between 925 and 975 °C (see Figure S1). This phase seems to be isostructural with the former oxycarbonate phase, but has a cell volume between BaCO_3_ and the phase observed at 1000 °C. Its intensity pattern can be fitted with the structural model derived from the 1000 °C measurement and lattice parameters of *a* = 6.129(1) Å, *c* = 7.698(2) Å (*V* = 83.5 Å ^3^ per *ABX*_3_ unit, *c/a* = 1.256). A more precise analysis of this higher-volume oxycarbonate phase from the high-temperature powder diffraction data is not possible, due to a strong overlap with reflections of Ba_2_TiO_4_ (see Figure 1). Its existence further supports the suggested topochemical nature of the reaction mechanism from a gradual decomposition of CO_3_^2–^ to CO_2_ and O^2–^ and the accompanied diffusion of TiO_2_ as Ti^4+^ and 2 O^2–^ into and CO_2_ out of the material, since its structural evolution indicates a carbonate-rich state closer to *β*-BaCO_3_.

It is necessary to address the limitations of our in situ diffraction study clearly at this point. In order to carry out the experiment, a specific heating procedure together with a choice of the measurement range has to be applied (see also Section 3.2). Only a limited number of reflections could be considered for the structural analysis reported here; furthermore, the high temperature leads to strong thermal displacement of the atoms, and the *B*_iso_ parameters correlate with site occupancies at the same time. Thus, it is not possible to determine precise site occupations of such a compositionally complex mixture from the data available. The structural model given in Figure 3 should be interpreted in the sense that the O_3–*x*_(CO_3_)_*x*_ sublattice is most likely strongly disordered. Assuming that the TiO_2_ diffusion as Ti^4+^ and 2 O^2–^ into the material is complete, one can however use the volume differences as an approximation for the amount of carbonate species still present in the material. An oxide ion has a space requirement of 15.4 Å^3^ (= ^4^/_3_·π·*r* ^3^(O^2–^)·0.74^–1^) in a close packing, while a carbonate ion possesses approximately three times this volume, i.e., 46.2 Å^3^ (the “C^4+^” ion fits into the triangular gap between three oxide ions and can be neglected). Considering the volume differences of the oxycarbonate phases, which are approximately 13.3 Å^3^ and 16.3 Å^3^ per *ABX*_3_ unit, we estimate that approximately up to one-ninth of the overall anions might be present as carbonate species in these phases, resulting in compositions of BaTiO_2.67–*d*_(CO_3_)_0.33+*d*_ for these previously unknown intermediate phases observed within the synthesis of BCZT.

We emphasize that the oxycarbonate phases identified in this work have not been reported before, although oxycarbonates were reported for other synthesis attempts of BaTiO_3_ using carbon-containing precursors or solvents. Oxycarbonate intermediate phases were reported in the literature for BaTiO_3_ prepared via aqueous chemical solution deposition (BaO_1–*x*_(CO_3_)_*x*_ ^17^), oxalate method (BaTiO_3–*x*_(CO_3_)_*x*_ ^26^), and from polymeric organic precursors (BaTiO_2.5_(CO_3_)_0.5_ ^27–30^ ). The previous reports have in common that the phases form at significantly lower temperatures, between 600 and 700 °C, and decompose just beyond this temperature; none of the reports provide a structural model or lattice parameters of the observed oxycarbonates, though the report from Ischenko et al.^28^ also observed a structure related to the one of *β*-BaCO_3_ from their diffraction data (note that a different intensity ratio of the pattern is shown and the phase was reported to have a low crystallinity). The reactants and reaction procedure used also imply that the way these materials are formed is different from that of trigonal BaTiO_2.67–*d*_(CO_3_)_0.33+*d*_ reported here. At least partial intermixing of Ba^2+^ and Ti^4+^ ions within an amorphous matrix of the precipitate containing O^2–^/OH^–^/H_2_O/CO_3_^2–^/C_2_O_4_^2–^ ions can be expected during solution-based synthesis with formation of a to-be-heated precipitate. Thus, these phases form via the decomposition of the anion matrix under the release of CO_2_/CO/H_2_O accompanied by crystallization, and the reactions are likely not of topochemical nature. Further, it is also remarkable that other in situ diffraction studies on the reaction of BaCO_3_ with TiO_2_ did not observe the trigonal perovskite-type oxycarbonates, indicating a crucial role of the way that the precursor is being prepared.^57^

The solid-state reaction mechanism for the formation of BaTiO_3_ from oxide and carbonate precursors has been widely investigated:^32,37^ The formation of the final BaTiO_3_ phase was discussed as a consecutive step after formation of an intermediate Ba_2_TiO_4_ phase formed by the reaction of TiO_2_ with BaCO_3_ (via 2 BaCO_3_ + TiO_2_ → BaTiO_3_ + BaCO_3_ + CO_2_ → Ba_2_TiO_4_ + 2 CO_2_), see Figure 4 (left). The fact that the high-temperature modification of BaCO_3_ provides a topochemical reaction route might be an important aspect for ceramic synthesis, since it would imply that microstructural aspects of *β*-BaCO_3_ (e.g., crystallite size) will at least partly influence the particulate nature of the targeted perovskite-type phase in the temperature range between 900 and 1000 °C (see Figure 4, right). This can be understood by considering other topochemical reactions observed for perovskite (-related) materials, for which the particle morphology before and after the reaction remains similar apart from the volume changes induced by compositional changes.^58^ This seems to be the case for the topochemical reaction route discussed in this article, as supported by the sharp reflections observed for *β*-BaCO_3_ (Figure 1, 850 °C), for the oxycarbonate phase (Figure 1, 1000 °C), and the perovskite phases formed on further temperature increase (in contrast to the broader perovskite reflections at e.g., 850 °C). Therefore, if one were able to limit the interdiffusion route via Ba_2_TiO_4_ by adjusting the heating conditions (e.g., by using fast-heating methods to reach the sweet-spot temperatures for the topochemical reaction), one could adjust the particle size distribution of the perovskite particles. The particle size distribution of the *β*-BaCO_3_ phase in the educt mixture could thus be used to indirectly influence the final sintering behavior, which strongly depends on the particle size, size distribution, and morphology. Since in applications the electroceramics are used in their dense/sintered form, this also decisively influences the material′s performance.^25^

Thus, we assume that the findings reported here could provide a future lever for optimizing the preparation conditions, which allow for more reliable microstructural engineering of ceramic materials. However, for deriving a detailed understanding of ratios of topochemical reaction route versus interdiffusion reaction and corresponding microstructural aspects, detailed studies based on a broad variation of microstructural aspects of the starting mixture (variation of milling time, preheating of precursors, etc.), possibly combined with in situ microscopic studies, would be required, which are beyond the scope of the current study. Beyond this, the extent to which the presence of Ca and Zr ions influences the adaptation for this reaction route could be addressed within subsequent studies.

One could also consider the topochemical reactivity of *β*-BaCO_3_ in the context of acidity and basicity of the reactants. Regarding this, the reaction behavior identified is in line with the following interpretation: in contrast to BaCO_3_, BaO is a very strong basic material. The high basicity of the oxide ions originates from the very different coordination chemistry within the compounds. Despite the different space group symmetry, both *β*-BaCO_3_ and BaO have an underlying substructure, which can be described by a close packing of Ba^2+^ ions, but the coordination of Ba^2+^ and thus the coordination of the anions is different. While BaO has a 6-fold octahedrally coordinated Ba^2+^ and O^2–^ ion species, *β*-BaCO_3_ enables a 9-fold coordination of Ba^2+^. However, for *β*-BaCO_3_, the oxide ions form a strong covalent bond to the carbon of the carbonate ion, and the further three additional bonds to Ba^2+^ weaken the basicity of the O^2–^ species significantly. In contrast, the six soft Ba^2+^ ions around O^2–^ in BaO result in weaker bonds and thus a higher basicity. The entropy-driven formation of oxide defects at high temperatures within *β*-BaCO_3_ according to

$$\beta -\mathrm{BaC}O_{3}\to {\mathrm{Ba}\left(CO_{3}\right)}_{1-d}O_{d}+dCO_{2}$$

will make the resulting O^2–^ defects in the material increasingly basic. Thus, the diffusion of Ti^4+^ into the material would energetically stabilize the oxide defects in *β*-BaCO_3_ by providing more covalent bonding.

Likewise, the acidity of Ba^2+^ in *β*-BaCO_3_ will increase at the same time once oxide defects upon CO_2_ release are formed, since it results in a lowering of the effective coordination number. Accordingly, Ba^2+^ ions in BaO are stronger electron acceptors (stronger Lewis acid), which additionally leads to the necessity of O^2–^ diffusion from TiO_2_ into the compound in order to stabilize the Ba coordination. This is also in accordance with previous studies,^59,60^ which analyzed the structural chemistry of Ba-based perovskites and have shown that the maximization of the Ba^2+^ coordination number is a strong driving force for the structural chemistry of these materials.

The authors emphasize that these structural arguments might also be in principle reflected in the different electronic structures of the materials *β*-BaCO_3_,^61^ BaO,^62^ and BaTiO_3_ ^63^ (see also Figures S5–S7), for which descriptions were found within the Materials Project.^64^ In some sense, the electronic structure of CO_2_-deficient *β*-BaCO_3_ might reveal interesting electronic features in addition to the more conceptual (structure-)­chemical discussion provided above. Nevertheless, the topochemical reactivity basically implies that the electronic structure of *β*-BaCO_3_ must transition via intermediate states to the final BaTiO_3_ electronic structure. Thus, the authors aim to address these relationships in more detail in subsequent work in order to understand the influence on the associated electronic structure changes^15^ on reactivities within topochemical reactions in more detail.

We also emphasize that we checked previous reports containing in situ diffraction data on the synthesis of related phases. Taking into account the limitations of visually inspecting powder patterns presented in previous studies, we suppose that similar oxycarbonate phases might have been reported previously, though they were thought to be *A*_2_ *B*O_4_-type phases such as BaCaTiO_4_ ^65^ or Ba_2_TiO_4_.^66^ However, many other studies show no indication for the formation of such a phase, highlighting the importance to describe the precursor sources and devices used in the best possible detail.

**Figure 4:**
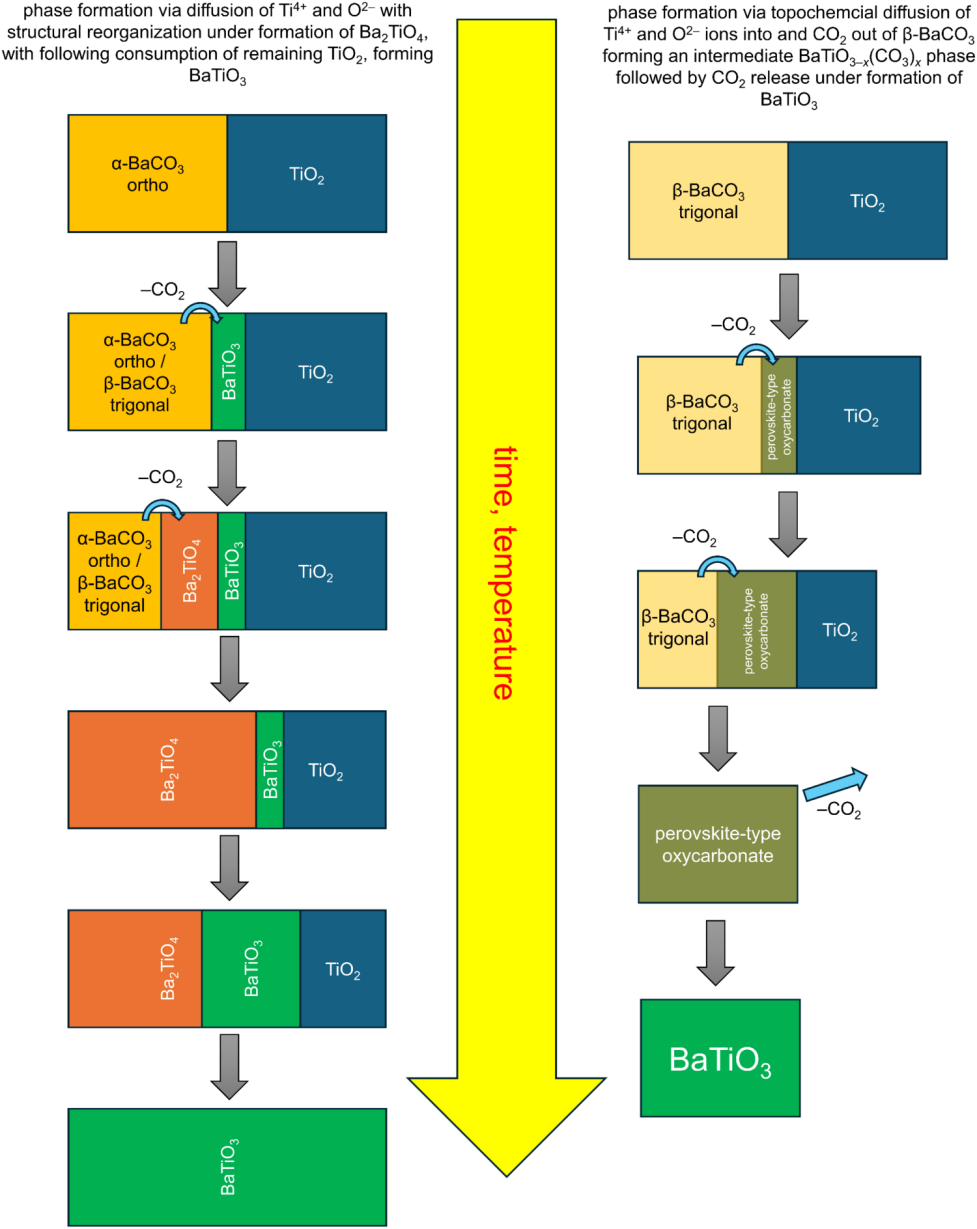
Comparison of reaction mechanisms reported for BaTiO_3_ via an intermediate Ba_2_TiO_4_ phase (left; according to Templeton and Pask^32^) in comparison to a topochemical diffusion of Ti^4+^ and 2 O^2–^ into *β*-BaCO_3_, accompanied by CO_2_ expulsion.

### 3.2 Stabilization of Trigonal Oxycarbonate Phases at Room Temperature through Quenching

Phases formed as intermediate reaction products at elevated temperature can often be quenched to ambient temperature, prohibiting further reaction. We performed various attempts in order to quench the reaction mixture from the temperature range 925–950 °C to ambient temperature to facilitate a more detailed structural analysis by recording synchrotron diffraction data (see Figure 5 with subsequent Rietveld analysis; Figure 6). We performed Raman spectroscopy measurements on a quenched powder sample to confirm that carbonate-containing phases are still present after quenching. The spectra shown in Figure S4 exhibit a broad feature between 1300 and 1700 cm^–1^, which is indicative of carbonate anions.^67^ Furthermore, we note that this feature is very broad, which may be caused by the convolution of various carbonate bands from oxycarbonate phases of different cell sizes and compositions. Indeed, we could observe perovskite-related modifications with significantly higher volume per formula unit than would be expected for BaTiO_3_ or BaZrO_3_. However, compared to the high-temperature in situ diffraction patterns shown in Figure 5, significantly higher amounts of oxide perovskites were observed. This indicates that further compositional and structural changes were induced on quenching, making the usability of this data for structural analysis limited. The oxycarbonate phases appear to the largest extent (visually evident from the area of the reflections in Figure 6) as broad bumps shifted to lower angles, as compared to the main BCZT phase. Additionally, a multitude of sharp reflections are superimposed on these broad bumps. These reflections could be identified to correspond to trigonal perovskite modifications (see later discussion). Nevertheless, we conclude that the simplicity of the pattern and the corresponding structure and composition, as observed at elevated temperatures via in situ diffraction, are not maintained after quenching. We also emphasize that a small amount of rutile-type TiO_2_ (∼4 wt.%) could be observed, which shows very sharp reflections.

**Figure 5:**
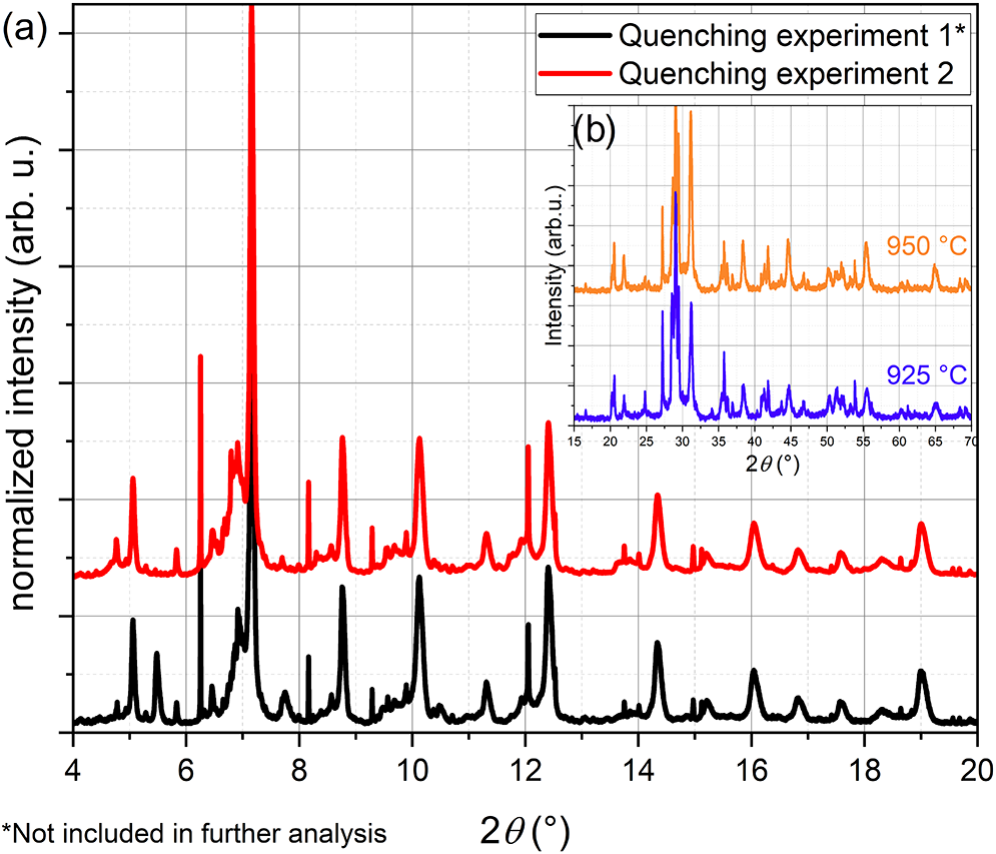
(a) Synchrotron diffraction data (λ = 0.35430(6) Å) of samples quenched from 925 °C after heating as described in Section 2. (b) In situ XRD data (Cu *K*_α_) at the quenching temperatures. The overall heating time of quenched sample 1 was reduced by an hour compared to sample 2 (which was used for further analysis).

**Figure 6:**
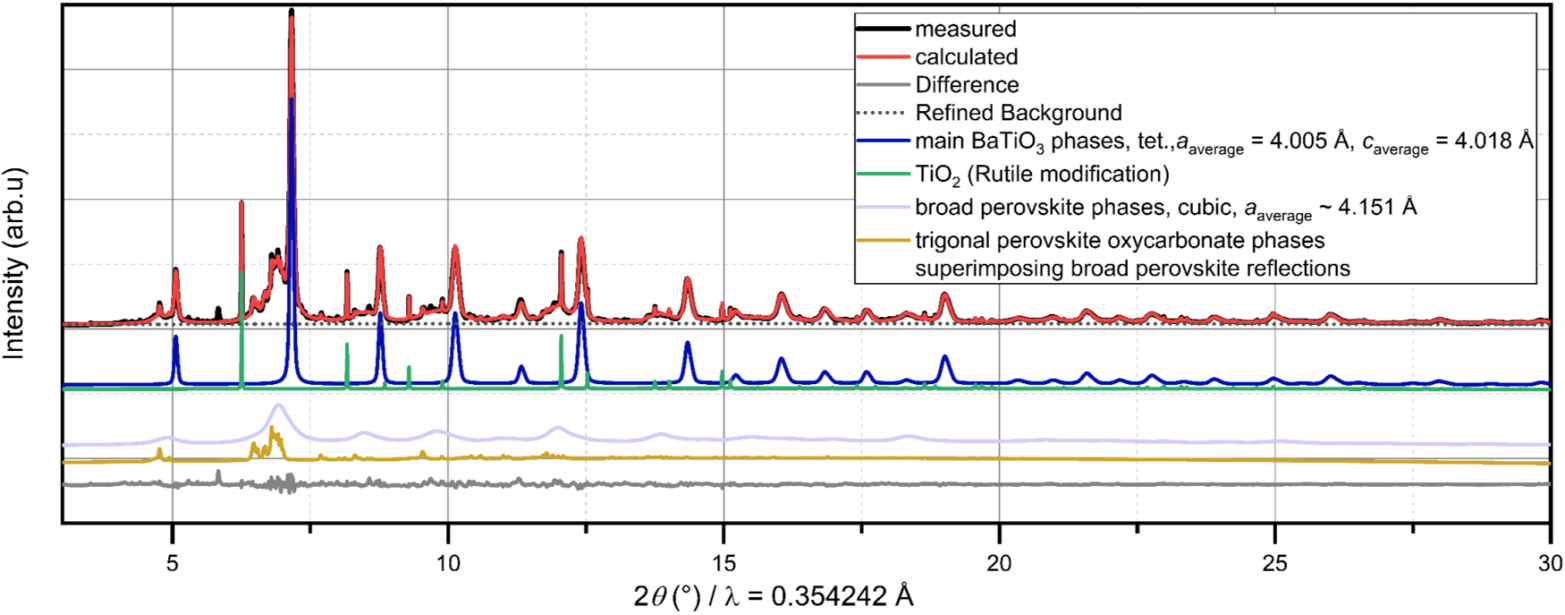
Rietveld analysis of synchrotron powder diffraction data (λ = 0.35430(6) Å) of a sample quenched from 925 °C to ambient temperature.

As discussed in the Supporting Information, the analysis of the sharp reflections superimposed on the broad perovskite reflections made it necessary to (1) describe the pattern of the broad underlying reflections precisely while (2) at the same time limiting the number of parameters. Though LeBail fits often can provide a description of broad reflections, we found that this already resulted in an unfavorable correlation. Thus, Rietveld models for the crystalline perovskite phases with sharp reflections (two tetragonal phases with slightly different lattice parameters identifying them as BaTiO_3_, see Figure 6) as well as for the low-crystallinity perovskite phase (overall described by three different perovskite phases with different lattice parameters) had to be used.

Based on this analysis, we found that although the reflection pattern around the (110)_cub._ reflection at ∼7° 2θ was highly complex, the pattern around the (100)_cub._ reflection was far simpler. From this, we could use constraints at the beginning of our analysis for the trigonal distortion in order to refine the observed reflection pattern, which were then relaxed afterward. The findings are depicted in Figure 7 and can be summarized as follows:

- Three trigonal phases are observed, which have a unit cell volume around 231 Å^3^, but differ with respect to the degree of trigonal distortion, with 5.96 Å ≥ *a* ≥ 5.87 Å and 7.51 Å ≤ *c* ≤ 7.73 Å (corresponding to *c*/*a*-ratios of 1.260–1.319 and being even larger than observed at higher temperature (for a pseudocubic trigonal setting a *c*/*a*-ratio of (3/2)^0.5^ = 1.225 would be expected)). From this, we conclude that on cooling, there might be a partial ordering of carbonate and oxide species taking place, which results in different degrees of structural distortions. We also observe that this phase has a similar volume difference per *ABX*_3_ formula unit of ∼13 Å^3^ to the tetragonal crystalline perovskite main phase, which is very similar to what has been observed at higher temperatures (13.3 Å^3^).
- Another trigonal phase is observed with a significantly smaller volume of 208.4 Å^3^. This phase has similar sharp reflections as the three other perovskite phases. The volume difference to one formula unit of BaTiO_3_ is much smaller and in the order of 5.5 Å^3^, which indicates that this phase might only contain 1/2–1/3 of the carbonate species. This is also consistent with the *c/a* ratio of this phase of ∼1.214, which is close to (3/2)^0.5^ = 1.225, and is in agreement with the observation made at high temperature that the smaller the carbonate content, the smaller the trigonal distortion.
- Additionally, a fifth trigonal phase could be identified. This phase shows very strong anisotropic broadening of the reflections, for which Stephens’ model^68^ had to be applied to describe the reflection shapes. This phase has a significantly larger cell volume of ∼241.6 Å^3^, corresponding to a difference of 16.5 Å^3^ per *ABX*_3_ unit with an even further increased *c*/*a* ratio of 1.335, both indicative of a higher carbonate content. Also here, the additional 3 Å^3^ volume increment is in line with what has been observed at higher temperature for the additional higher-volume oxycarbonate phase at 925–975 °C. The anisotropic strain broadening might further be explained by increased chemical strain on disorder within the *ab*-plane (reflections with index (001) are significantly sharper) due to the increased carbonate content.

**Figure 7:**
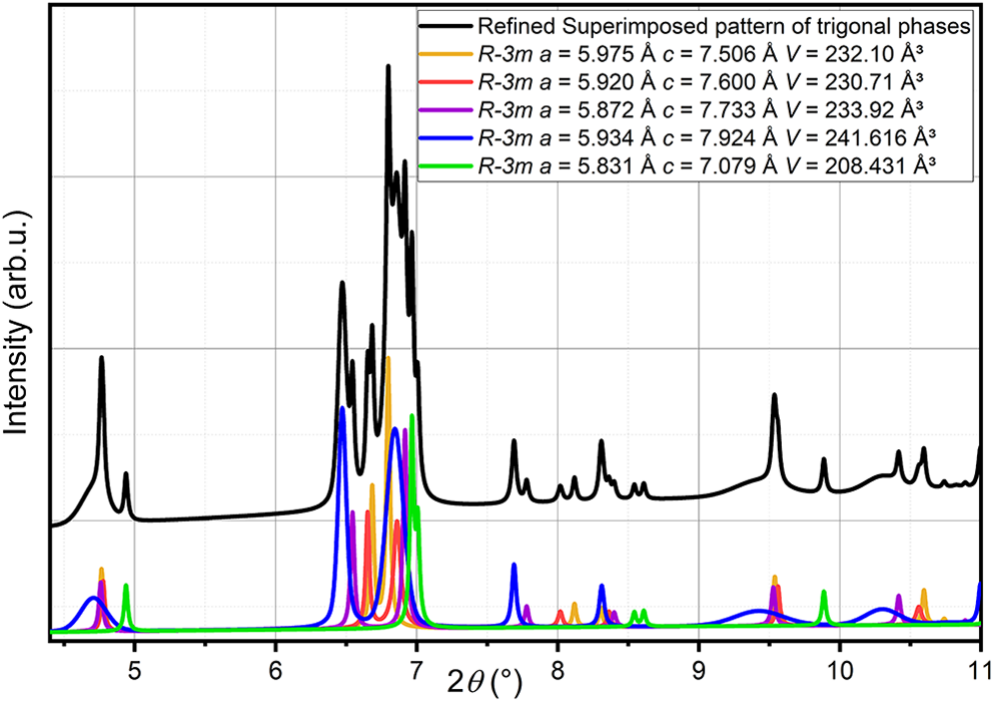
Individual trigonal oxycarbonate phases identified on a sample quenched to ambient temperature. Detailed refinement results are provided in the Supporting Information. We emphasize that the sum of the colored curves is shown as a black curve for better visibility (which is identical to the purple curve in Figure 6).

Due to the fact that the powder diffraction pattern becomes even more complicated on quenching, with large reflection overlap and reflections superimposed on broad bumps, a more detailed analysis of positional parameters or compositions was not stable. Thus, we used a single structural model for all five trigonal phases, which indicated structural disorder from the high thermal parameters observed for both *A*- and *B*-site cations (see Supporting Information). However, we would like to make the reader aware that these structural data should not be overinterpreted due to the extremely high correlation observed and the fact that the intensity of the broad underlying bumps could only be refined approximately. However, since we could verify the presence of all reflections including an approximate intensity pattern, and since the cell volumes correlate well with what was observed for the high-temperature measurements, we conclude that these phases can be in principle quenched to room temperature. For transparency reasons, we provide the refinement file in the Supporting Information, acknowledging that the synchrotron data still indicate additional reflections, especially visible when applying a logarithmic scaling, as well as from the difference curve shown in Figure 6. Attempts to assign these phases to (e.g., 2 × 2 × 2) superstructures of the trigonal oxycarbonates did not prove to be successful; thus, we currently assume that these are likely corresponding to additional unknown Ba-Ca-Ti-Zr-O-CO_3_ phases.

The performed Rietveld analysis also allows us to roughly quantify the different phases present at room temperature. From this, it is indicated that approximately 42 wt.% of the sample is present as tetragonal BCZT, 40 wt.% is present as broad perovskite phases with an approximate lattice parameter of 4.14 Å, and 14 wt.% is present as trigonal oxycarbonate phases. Additionally, the sample contains 4 wt.% of TiO_2_ and small amounts of impurity phases (presumably less than ∼3 wt.% from the intensity misfit of the difference curve).

We also undertook efforts to determine the crystal structure of a trigonal phase using 3D electron diffraction (ED) data collected under cryogenic conditions (Figure 8). The data could be indexed with a trigonal unit cell characterized by parameters *a* = 5.74 Å, *c* = 6.97 Å, and γ = 120°. Metrical symmetry analysis, performed using the LEPAGE program,^69^ confirmed their consistency with trigonal symmetry. The initial structure was solved in space group *R*3̅*m* (No. 166). Refinements against the 3D ED data converged with an *R*_1_ value of 17.89%. The position of the (CO_3_)^2–^ anion was not refined due to the small unit cell volume (198.9 Å^3^), which suggests the presence of a carbonate-deficient variant. The crystallite measured approximately 170 nm in size. Incorporation of Ca and Zr cations at the *A*- and *B*-sites resulted in full occupancy of Ba and Ti. This adjustment did not improve the refinements.

These findings are consistent with our analysis of the synchrotron powder diffraction data. The *c/a*-ratio of 1.214, obtained from ED, is virtually identical to that of the oxycarbonate phase with the smallest volume, identified from the synchrotron diffraction data. Considering that the ED data were collected on a comparatively small powder particle (Figure 8a; 170 nm compared to the average particle size of the precursor mixture of 960 nm; see also Figure S8), finding the most carbonate-deficient variant is consistent with our hypothesis. Since we assume that oxycarbonate intermediate phases decompose into the *AB*O_3_ perovskite matrix phase via the expulsion of CO_2_ into the atmosphere (right mechanism in Figure 4), we would expect smaller oxycarbonate particles to decompose into carbonate-deficient variants more quickly than larger ones, due to the significantly shorter diffusion pathways for C^4+^ and O^2–^. We note that the ADF-STEM analysis of the powder sample revealed pronounced morphological variation in the crystallites (see Figure S8), and the analyzed oxycarbonate crystallite does not represent a “typical” particle in terms of its specific morphology (smaller than 200 nm with a rounded shape and jagged edges). Based on our previous investigation, it is expected that transient perovskite phases, residual precursors, fully formed BCZT, and Ba_2_TiO_4_ intermediates are also present in the sample at the quenching temperature of 925 °C.^31,32^ Due to the pronounced compositional inhomogeneity within the oxycarbonate phases identified in the quenched state (see Figure 7) and the similarity to the perovskite matrix, unambiguous phase identification is only possible using ED on very small particles (like the analyzed particle marked in Figure 8a). A detailed morphological analysis of a large number of representative oxycarbonate particles is thus not possible with the applied methods in the quenched state.

**Figure 8:**
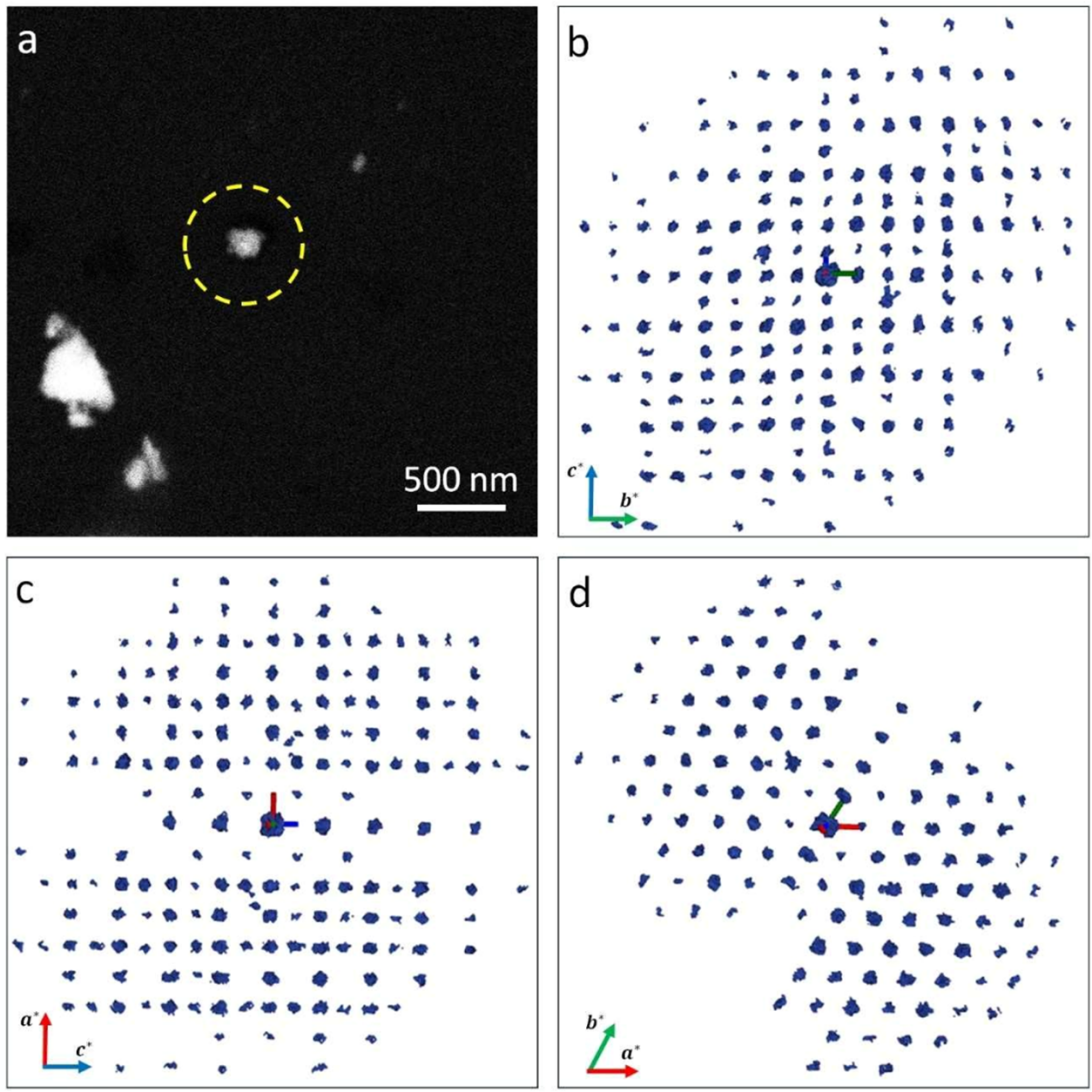
(a) STEM image of the measured crystallite. (b–d) 3D reciprocal lattice of the trigonal phase reconstructed from a 3D ED data set showing the (b) 0kl, (c) h0l, and (d) hk0 projections.

## 4 Conclusions

In this article, we have shown that the phase transformation of BaCO_3_ to its trigonal calcite-related high-temperature modification provides a reaction pathway for the formation of BaTiO_3_ via the incorporation of Ti^4+^ and 2O^2–^ within a topochemical reaction, while CO_2_ is expelled. Interestingly, the calcite modification of CaCO_3_ ^70^ is structurally related and SrCO_3_ has an isotypic high-temperature modification to *β*-BaCO_3_;^71^ however, a similar reaction behavior of these materials has not been reported for perovskite synthesis. Therefore, the increased volume for the Ba-homologue might be a prerequisite in order to facilitate the topochemical reaction pathway for perovskite syntheses. Nevertheless, the reaction behavior for other alkaline earth (*AE*) containing, possibly via preparation of multication Ba_1–x_ *AE*_x_CO_3_ materials, has not been studied, and thus might provide a pathway to facilitate this reaction behavior as well.

## Supplementary Material



## Notes

In situ diffractogram, showing all identified phases in the BCZT precursor powder mixture at 925 °C (Figure S1); schematics and simulated diffractograms, illustrating the relationship between a cubic perovskite phase and the *R*3̅*m* oxycarbonate phase (Figures S2 and S3); full Rietveld analysis report for the synchrotron diffractogram of a BCZT precursor mixture quenched from 925 °C to room temperature; Raman spectra and ADF-STEM images of a quenched precursor powder mixture (Figures S5 and S8); band structures and pDOS of BaTiO_3_, BaO_2_, and BaCO_3_ taken from the Materials Project (PDF)
