# Supplementary material for: β‑BaCO3 Facilitates a Topochemical Reaction Route for the High-Temperature Synthesis of Perovskite-Type (Ba,Ca)(Zr,Ti)O3 via Oxycarbonate Intermediates
Source: Chem Mater. 2026 Apr 2;38(7):3449–61. doi: 10.1021/acs.chemmater.5c03257 (PMC13084997; doi:10.1021/acs.chemmater.5c03257)
Supplement: Supplementary file 1 [file cm5c03257_si_001.pdf]

Supplementary Information for

β-$BaCO_3$ facilitates a topochemical reaction route for the high temperature synthesis of perovskite-type $(Ba,Ca)(Zr,Ti)O_3$ via oxycarbonate intermediates

Anna M. Paulik[a], Marc Widenmeyer[b], Sylvia L. Kunz[c], Magdalena O. Cichocka[d], Ute Kolb[d], Jurij Koruza[a,*], Oliver Clemens[c,*]

[a] Institute for Chemistry and Technology of Materials, Graz University of Technology, Stremayrgasse 9, 8010 Graz, Austria

[b] Technical University of Darmstadt, Institute for Materials Science, Materials and Resources, Peter-Grünberg-Straße 2, 64287 Darmstadt, Germany

[c] University of Stuttgart, Institute for Materials Science, Materials Synthesis Group, Heisenbergstraße 3, 70569 Stuttgart, Germany

[d] Technical University of Darmstadt, Institute of Applied Geosciences, Electron Crystallography Group, Schnittspahnstraße 9, 64287 Darmstadt, Germany

* Corresponding Authors:

Prof. Dr. Oliver Clemens	Assoc. Prof. Dr. Jurij Koruza
Email: oliver.clemens@imw.uni-stuttgart.de	jurij.koruza@tugraz.at
Fax: +49 711 685 51933	+43 316 873 32305

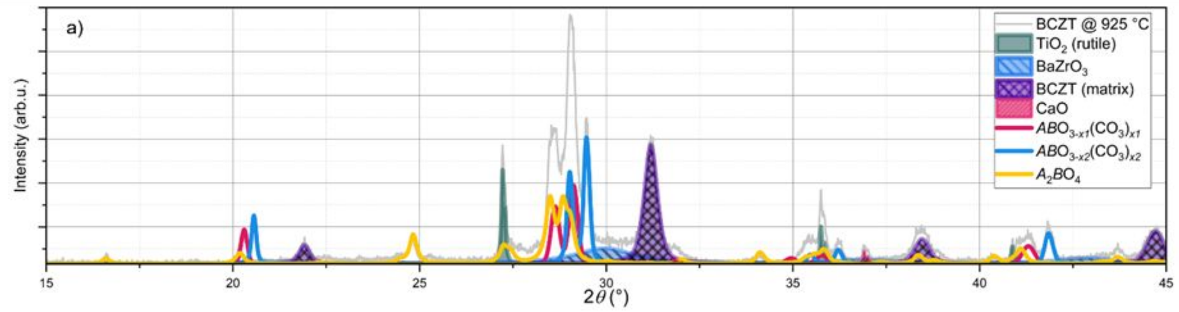

Figure S1. *In-situ diffraction pattern of BCZT at 925 °C, showing that the additional intensity observed in the range of 27° ≤ 2θ ≤ 30° which can only be partially explained by the formation of Ba_2TiO_4 (A_2BO_4, yellow partial curve).*

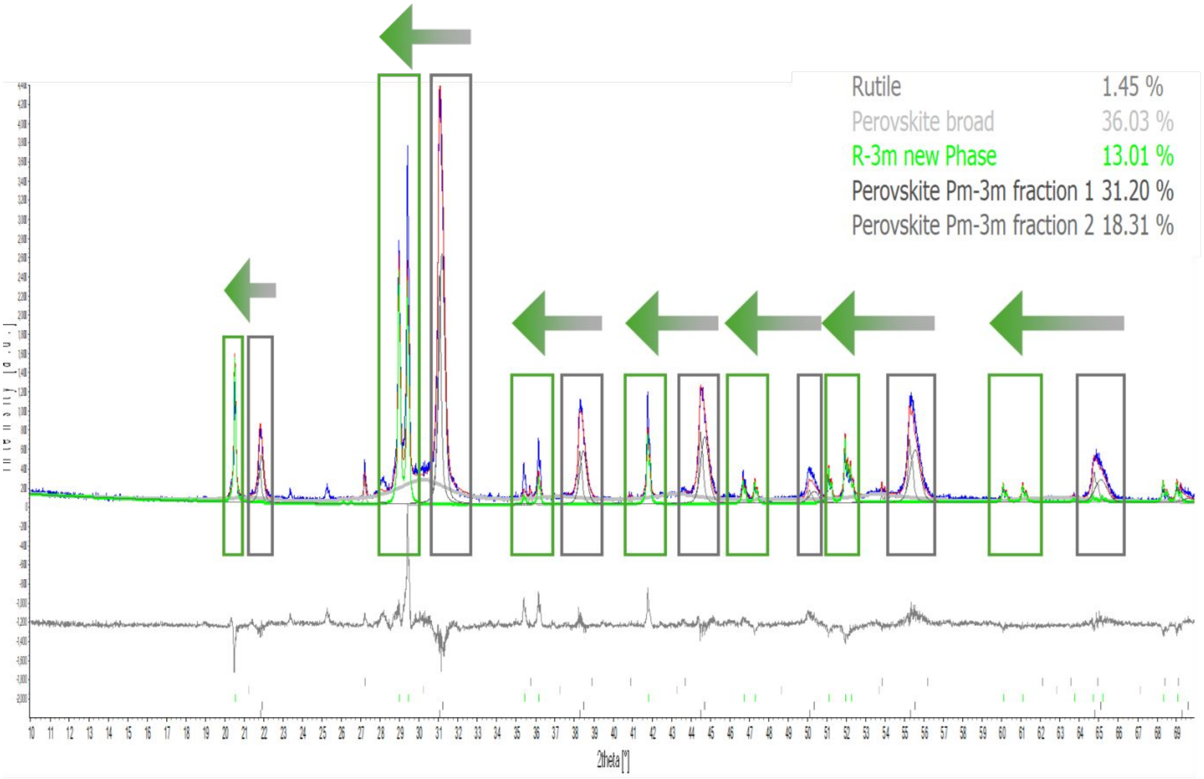

Figure S2. *Fit of the in-situ diffraction pattern obtained for the synthesis of BCZT at 1000 °C. The similarity of both, the intensity and the positional pattern is indicated from comparing the Rietveld fits of the perovskite-related trigonal ($R\overline{3}m$) phase (green) to the main perovskite-type phases contained in the pattern (gray). For the fit shown here, only Ba (3a) and Ti (3b) were used in a perovskite-like lattice within the $R\overline{3}m$ unit cell.*

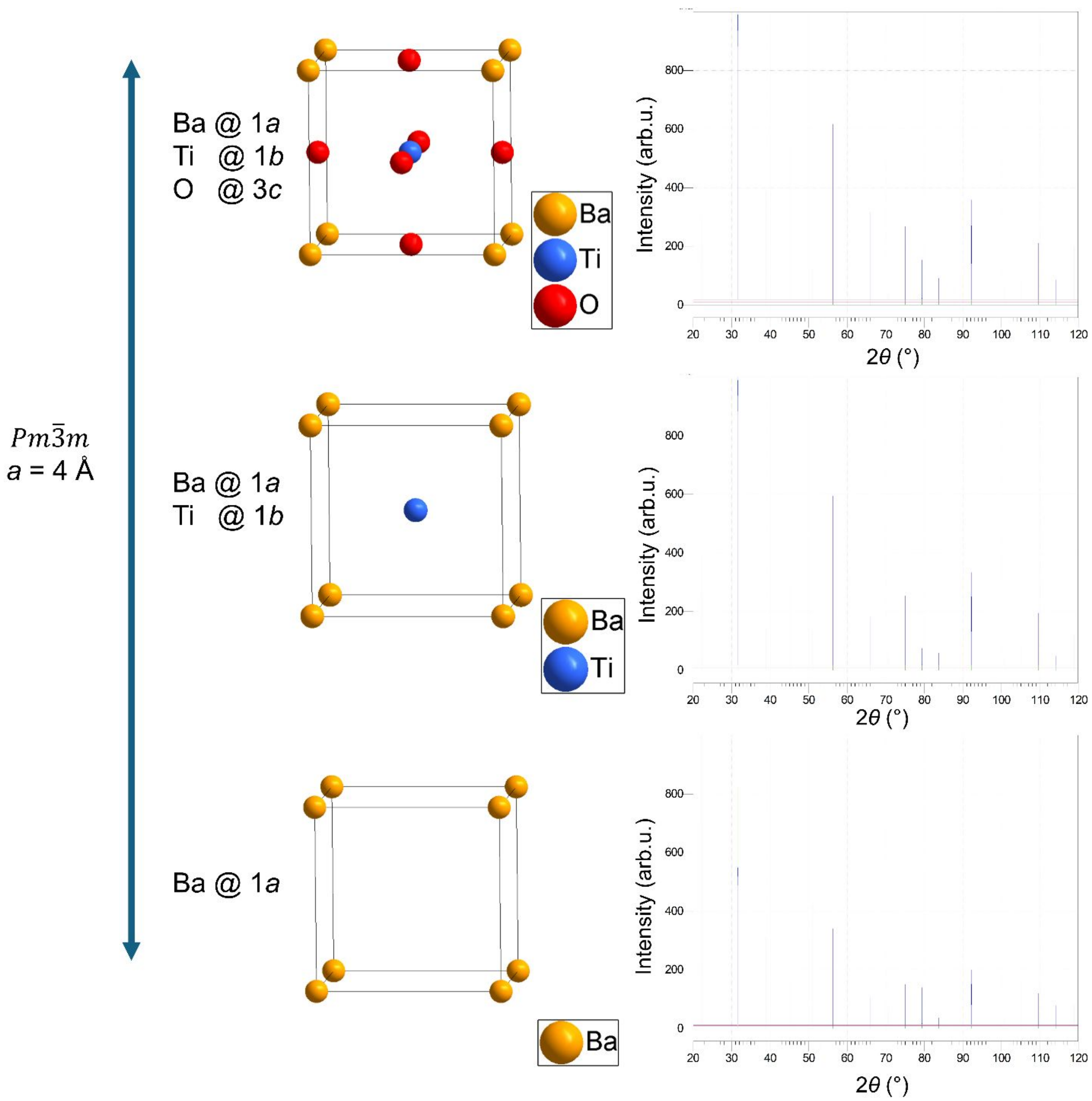

Figure S3. *Diffraction patterns of cubic perovskite* $BaTiO_3$ *and a hypothetical structurally related CsCl-type BaTi and α-Po-type Ba. The diffraction patterns of* $BaTiO_3$ *and BaTi are similar (though not identical) due to the fact that the oxygen ions are the weakest scatterers and thus have a comparably low contribution to the overall intensity pattern. In contrast, Ba has a significantly different intensity pattern compared to BaTi and* $BaTiO_3$*.*

Analysis Report for Rietveld-Analysis of Synchrotron Diffraction Data of a sample quenched from 925 °C to ambient temperature

Analysis Report

Data Files

Data file 1 : ch7369_Oxy-925C_295K_w0003_b0001.xye

Global R-Values

Rexp : 3.14	Rwp : 6.81	Rp : 4.59	GOF : 2.17
Rexp`: 4.67	Rwp`: 10.13	Rp` : 7.30	DW : 0.06

File 1 : "ch7369_Oxy-925C_295K_w0003_b0001.xye"

Range Number : 1

R-Values

Rexp : 3.14	Rwp : 6.81	Rp : 4.59	GOF : 2.17
Rexp`: 4.67	Rwp`: 10.13	Rp` : 7.30	DW : 0.06

Quantitative Analysis - Rietveld

Phase 1	: Rutile	4.084 %
Phase 2	: "R-3m a = 5.975 c = 7.506 V = 232.10"	2.303 %
Phase 3	: "Tetragonal sharp1 a = 4.012 c = 4.024"	27.801 %
Phase 4	: "Tetragonal sharp2 a = 3.991 c = 4.018"	14.041 %
Phase 5	: "R-3m a = 5.920 c = 7.600 V = 230.71"	1.853 %
Phase 6	: "R-3m a = 5.872 c = 7.733 V = 233.92"	1.917 %
Phase 7	: "R-3m a = 5.934 c = 7.924 V = 241.616"	6.453 %
Phase 8	: "broad perovskite a = 4.154"	31.984 %
Phase 9	: "broad perovskite a = 4.029"	5.876 %
Phase 10	: "broad perovskite a = 4.444"	2.016 %
Phase 11	: "R-3m a = 5.831 c = 7.079 V = 208.431"	1.674 %

**Degree of crystallinity (%)**	63.39
Crystalline area	11309.412
Amorphous area	6530.393

Background

Chebychev polynomial, Coefficient	0	364.3807
	1	-1.7889
	2	-40.1871

Instrument

Primary radius (mm)	217.5
Secondary radius (mm)	217.5
Receiving slit width (mm)	0.007144731
FDS angle (°)	0.0001
Tube_Tails	
Source Width (mm)	0.007082964
Z1 (mm)	-1
Z2 (mm)	1
Fraction	5e-008

Corrections

Zero error	0
LP Factor	90

Miscellaneous

X Calculation Step	0.001
Start X	1.8
Finish X	30

Structure 1

Phase name	Rutile
R-Bragg	2.784
Spacegroup	136
Scale	1.75704e-005
Cell Mass	319.463
Cell Volume (Å^3)	62.40745
Wt% - Rietveld	4.084
Double-Voigt|Approach	
Cry size Lorentzian	740.1
Cry size Gaussian	1494.6
k: 1 LVol-IB (nm)	414.415
k: 0.89 LVol-FWHM (nm)	542.989
Strain	
Strain L	0.0590051
Strain G	0.06695355
e0	0.00023
Crystal Linear Absorption Coeff. (1/cm)	15.503
Crystal Density (g/cm^3)	8.500
Lattice parameters	
a (Å)	4.5924051
c (Å)	2.9590756

Site	Np	x	y	z	Atom	Occ	Beq
Ti1	2	0.00000	0.00000	0.00000	Ti+4	1	0.6627
O1	4	0.30570	0.30570	0.00000	O-2	1	0.6627
Ti1	2	0.00000	0.00000	0.00000	Ti+4	1	0.6627
O1	4	0.30450	0.30450	0.00000	O-2	1	0.6627

Structure 2

Phase name	R-3m a = 5.975 c = 7.506 V = 232.10
R-Bragg	2.509
Spacegroup	R-3m
Scale	1.23297e-006
Cell Mass	690.237
Cell Volume (Å^3)	232.09631
Wt% - Rietveld	2.303
Double-Voigt|Approach	
Cry size Lorentzian	69.5
k: 1 LVol-IB (nm)	44.270
k: 0.89 LVol-FWHM (nm)	61.890
Crystal Linear Absorption Coeff. (1/cm)	21.845
Crystal Density (g/cm^3)	4.938
Lattice parameters	
a (Å)	5.9752149
c (Å)	7.5063801

Site	Np	x	y	z	Atom	Occ	Beq
Ba1	3	0.00000	0.00000	0.00000	Ba+2	1	7
Ti1	3	0.00000	0.00000	0.50000	Ti+4	1	7
C	9	0.50000	0.00000	0.00000	O	0.2672	-0.3884
O1	6	0.33333	0.66667	0.00000	O	0.501	-0.3884
O2	6	0.66667	0.33333	0.00000	O	0.501	-0.3884

Structure 3

```
   Phase name                              Tetragonal sharp1 a = 4.012 c
= 4.024
   R-Bragg                                 1.256
   Spacegroup                              P4/mmm
   Scale                                     1.64940e-004
   Cell Mass                               223.162
   Cell Volume (Å^3)                       64.78328
   Wt% - Rietveld                          27.801
   Double-Voigt|Approach
      Cry size Lorentzian                  100.0
      k:  1  LVol-IB (nm)                  63.662
      k:  0.89  LVol-FWHM (nm)             89.000
   Strain
      Strain G                             0.3127623
      e0                                   0.00068
   Crystal Linear Absorption Coeff. (1/cm) 24.763
   Crystal Density (g/cm^3)                5.720
   Lattice parameters
      a (Å)                                4.0121354
      c (Å)                                4.0244983
```

Site	Np	x	y	z	Atom	Occ	Beq
Ba1	1	0.00000	0.00000	0.00000	Ba+2	0.8853	0.6627
					Ca+2	0.1147	0.6627
Zr1	1	0.50000	0.50000	0.50000	Ti+4	0.9743	0.6627
					Zr+4	0.02569	0.6627
O1	1	0.50000	0.50000	0.00000	O-2	1	0.6627
""	2	0.00000	0.50000	0.50000	O-2	1	0.6627

Structure 4

```
   Phase name                              Tetragonal sharp2 a = 3.991 c
= 4.018
   R-Bragg                                 1.233
   Spacegroup                              P4/mmm
   Scale                                     8.43278e-005
   Cell Mass                               223.158
   Cell Volume (Å^3)                       63.99866
   Wt% - Rietveld                          14.041
   Double-Voigt|Approach
      Cry size Lorentzian                  100.0
      k:  1  LVol-IB (nm)                  63.662
      k:  0.89  LVol-FWHM (nm)             89.000
   Strain
      Strain G                             0.3127623
      e0                                   0.00068
   Crystal Linear Absorption Coeff. (1/cm) 25.066
   Crystal Density (g/cm^3)                5.790
   Lattice parameters
      a (Å)                                3.9909789
```

c (Å)	4.0180194

Site	Np	x	y	z	Atom	Occ	Beq
Ba	1	0.00000	0.00000	0.00000	Ba	0.9599	0.6627
Ca					Ca	0.04013	0.6627
Zr	1	0.50000	0.50000	0.50000	Zr	3.093e-035	0.6627
Ti					Ti	1	0.6627
O	3	0.50000	0.50000	0.00000	O	1	0.6627

Structure 5

Phase name	R-3m a = 5.920 c = 7.600 V = 230.71
R-Bragg	2.475
Spacegroup	R-3m
Scale	1.00000e-006
Cell Mass	688.864
Cell Volume (Å^3)	230.71482
Wt% - Rietveld	1.853
Double-Voigt|Approach	
Cry size Lorentzian	69.6
k: 1 LVol-IB (nm)	44.314
k: 0.89 LVol-FWHM (nm)	61.951
Crystal Linear Absorption Coeff. (1/cm)	21.973
Crystal Density (g/cm^3)	4.958
Lattice parameters	
a (Å)	5.9204780
c (Å)	7.6003103

Site	Np	x	y	z	Atom	Occ	Beq
Ba1	3	0.00000	0.00000	0.00000	Ba+2	1	7
Ti1	3	0.00000	0.00000	0.50000	Ti+4	1	7
C	9	0.50000	0.00000	0.00000	O	0.2672	-0.3884
O1	6	0.33333	0.66667	0.00000	O	0.501	-0.3884
O2	6	0.66667	0.33333	0.00000	O	0.501	-0.3884

Structure 6

Phase name	R-3m a = 5.872 c = 7.733 V = 233.92
R-Bragg	4.487
Spacegroup	R-3m
Scale	1.02050e-006
Cell Mass	688.864
Cell Volume (Å^3)	233.91601
Wt% - Rietveld	1.917
Double-Voigt|Approach	
Cry size Lorentzian	65.5
k: 1 LVol-IB (nm)	41.691
k: 0.89 LVol-FWHM (nm)	58.285
Crystal Linear Absorption Coeff. (1/cm)	21.672
Crystal Density (g/cm^3)	4.890
Lattice parameters	
a (Å)	5.8722637
c (Å)	7.8328215

Site	Np	x	y	z	Atom	Occ	Beq
Ba1	3	0.00000	0.00000	0.00000	Ba+2	1	7
Ti1	3	0.00000	0.00000	0.50000	Ti+4	1	7

C	9	0.50000	0.00000	0.00000	O	0.2672	-0.3884
O1	6	0.33333	0.66667	0.00000	O	0.501	-0.3884
O2	6	0.66667	0.33333	0.00000	O	0.501	-0.3884

Structure 7

Phase name	R-3m a = 5.934 c = 7.924 V = 241.616
R-Bragg	1.819
Spacegroup	R-3m
Scale	3.32535e-006
Cell Mass	688.864
Cell Volume (Å^3)	241.61604
Wt% - Rietveld	6.453
Double-Voigt|Approach	
Cry size Lorentzian	57.1
k: 1 LVol-IB (nm)	36.325
k: 0.89 LVol-FWHM (nm)	50.783
Crystal Linear Absorption Coeff. (1/cm)	20.981
Crystal Density (g/cm^3)	4.734
Lattice parameters	
a (Å)	5.9336313
c (Å)	7.9241740

Site	Np	x	y	z	Atom	Occ	Beq
Ba1	3	0.00000	0.00000	0.00000	Ba+2	1	7
Ti1	3	0.00000	0.00000	0.50000	Ti+4	1	7
C	9	0.50000	0.00000	0.00000	O	0.2672	-0.3884
O1	6	0.33333	0.66667	0.00000	O	0.501	-0.3884
O2	6	0.66667	0.33333	0.00000	O	0.501	-0.3884

Structure 8

Phase name	broad perovskite a = 4.154
R-Bragg	1.057
Spacegroup	221
Scale	1.66836e-004
Cell Mass	229.303
Cell Volume (Å^3)	71.71069
Wt% - Rietveld	31.984
Double-Voigt|Approach	
Cry size Lorentzian	5.5
Cry size Gaussian	10000.0
k: 1 LVol-IB (nm)	3.498
k: 0.89 LVol-FWHM (nm)	4.889
Strain	
Strain L	0.0001
Strain G	1.812387
e0	0.00395
Crystal Linear Absorption Coeff. (1/cm)	22.868
Crystal Density (g/cm^3)	5.310
Lattice parameters	
a (Å)	4.1545880
Degree of crystallinity status	Amorphous

Site	Np	x	y	z	Atom	Occ	Beq
Ba	1	0.00000	0.00000	0.00000	Ba	0.9599	0.6627
Ca					Ca	0.04013	0.6627
Zr	1	0.50000	0.50000	0.50000	Zr	3.093e-035	0.6627

Ti					Ti	1	0.6627
O	3	0.50000	0.50000	0.00000	O	1	0.6627

Structure 9

Phase name	broad perovskite a = 4.029
R-Bragg	0.788
Spacegroup	Pm-3m
Scale	3.36152e-005
Cell Mass	229.257
Cell Volume (Å^3)	65.39950
Wt% - Rietveld	5.876
Double-Voigt|Approach	
Cry size Lorentzian	5.5
Cry size Gaussian	10000.0
k: 1 LVol-IB (nm)	3.498
k: 0.89 LVol-FWHM (nm)	4.889
Strain	
Strain L	0.0001
Strain G	1.812387
e0	0.00395
Crystal Linear Absorption Coeff. (1/cm)	25.067
Crystal Density (g/cm^3)	5.821
Lattice parameters	
a (Å)	4.0289462

Site	Np	x	y	z	Atom	Occ	Beq
Ba	1	0.00000	0.00000	0.00000	Ba	0.9599	0.6627
Ca					Ca	0.04013	0.6627
Zr	1	0.50000	0.50000	0.50000	Zr	3.093e-035	0.6627
Ti					Ti	1	0.6627
O	3	0.50000	0.50000	0.00000	O	1	0.6627

Structure 10

Phase name	broad perovskite a = 4.444
R-Bragg	1.884
Spacegroup	Pm-3m
Scale	8.59172e-006
Cell Mass	229.257
Cell Volume (Å^3)	87.76855
Wt% - Rietveld	2.016
Double-Voigt|Approach	
Cry size Lorentzian	5.5
Cry size Gaussian	10000.0
k: 0.89 LVol-IB (nm)	3.113
k: 0.89 LVol-FWHM (nm)	4.889
Strain	
Strain L	0.0001
Strain G	1.812387
e0	0.00395
Crystal Linear Absorption Coeff. (1/cm)	18.679
Crystal Density (g/cm^3)	4.337
Lattice parameters	
a (Å)	4.4440573

Site	Np	x	y	z	Atom	Occ	Beq
Ba	1	0.00000	0.00000	0.00000	Ba	0.9599	0.6627
Ca					Ca	0.04013	0.6627

Zr	1	0.50000	0.50000	0.50000	Zr	3.093e-035	0.6627
Ti					Ti	1	0.6627
O	3	0.50000	0.50000	0.00000	O	1	0.6627

Structure 11

Phase name	R-3m a = 5.831 c = 7.079 V = 208.431
R-Bragg	3.764
Spacegroup	R-3m
Scale	1.00000e-006
Cell Mass	688.864
Cell Volume (Å^3)	208.43153
Wt% - Rietveld	1.674
Double-Voigt|Approach	
Cry size Lorentzian	69.5
k: 0.89 LVol-IB (nm)	39.400
k: 0.89 LVol-FWHM (nm)	61.890
Crystal Linear Absorption Coeff. (1/cm)	24.322
Crystal Density (g/cm^3)	5.488
Lattice parameters	
a (Å)	5.8308858
c (Å)	7.0788664

Site	Np	x	y	z	Atom	Occ	Beq
Ba1	3	0.00000	0.00000	0.00000	Ba+2	1	7
Ti1	3	0.00000	0.00000	0.50000	Ti+4	1	7
C	9	0.50000	0.00000	0.00000	O	0.2672	-0.3884
O	6	0.33333	0.66667	0.00000	O	0.501	-0.3884
O	6	0.66667	0.33333	0.00000	O	0.501	-0.3884

Comments on the analysis of synchrotron diffraction data: The thermal displacement parameters Beq were constrained to be identical for all atoms of the crystalline oxide perovskite phases as well as the broad perovskite phases; they partly turn negative, which is not physical but results from the absorption of Ba-containing materials, which weakens the reflections at lower angles stronger and thus "mimicks" a negative Beq value; in addition, it partly correlates with the occupation of this site. A- and B-site cations (7 Å²). All trigonal perovskite phases were constrained to be structurally identical, allowing only variation of the lattice parameters as well as partially different broadening of the reflections. A polynomial of order two had to be used to refine the background. Instrumental broadening functions were provided by the instrument scientists.

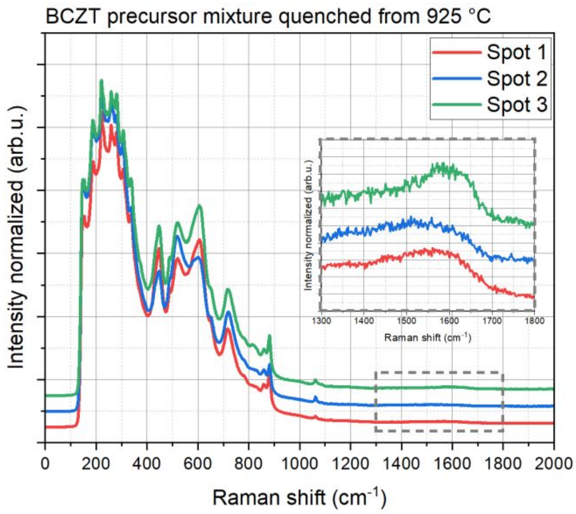

***Figure S4**. Raman spectra of a BCZT precursor mixture, quenched from 925 °C, measured on three different locations on the sample at RT. The most characteristic absorption band of an isolated planar CO_3^{2-} anion is expected to be observed around 1449 cm^{-1} [1], which is within the highlighted region, where a broad signal was recorded from 1300-1700 cm^{-1}.*

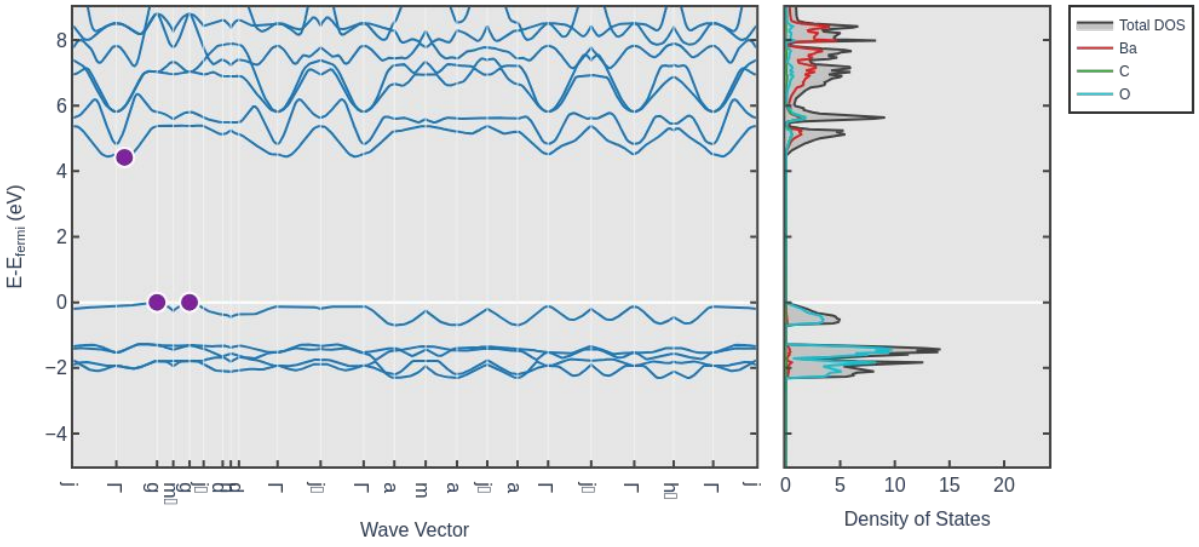

***Figure S5**. Band Structure and DOS / pDOS of $BaCO_3$[2-4]; image taken from [5] (licensed under CC BY 4.0).*

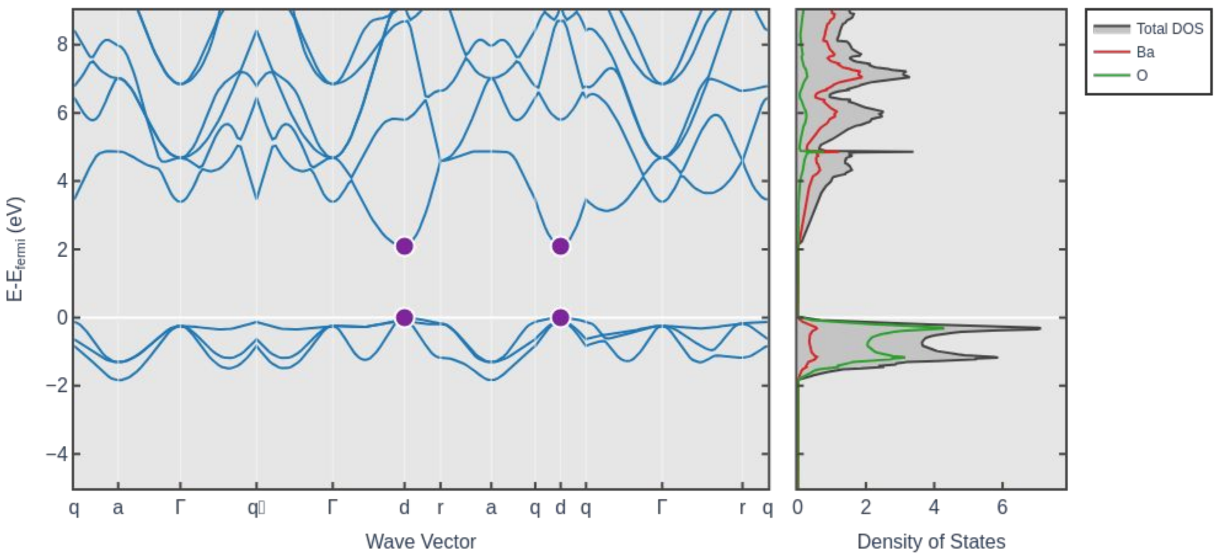

Figure S6. *Band Structure and DOS / pDOS of BaO^{2-4}; image taken from [6] (licensed under CC BY 4.0).*

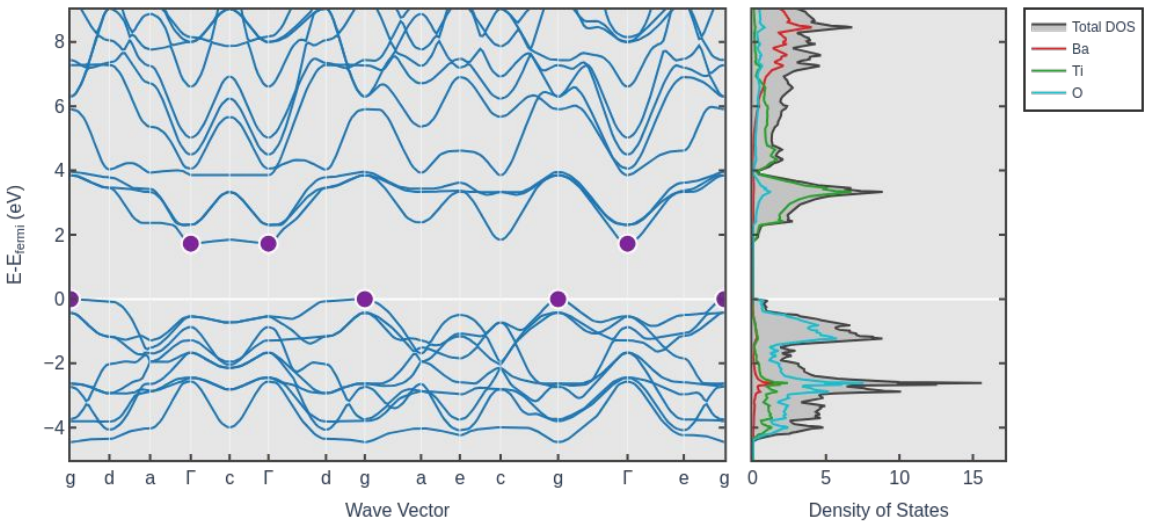

Figure S7. *Band Structure and DOS / pDOS of $BaTiO_3^{2-4}$; image taken from [7] (licensed under CC BY 4.0).*

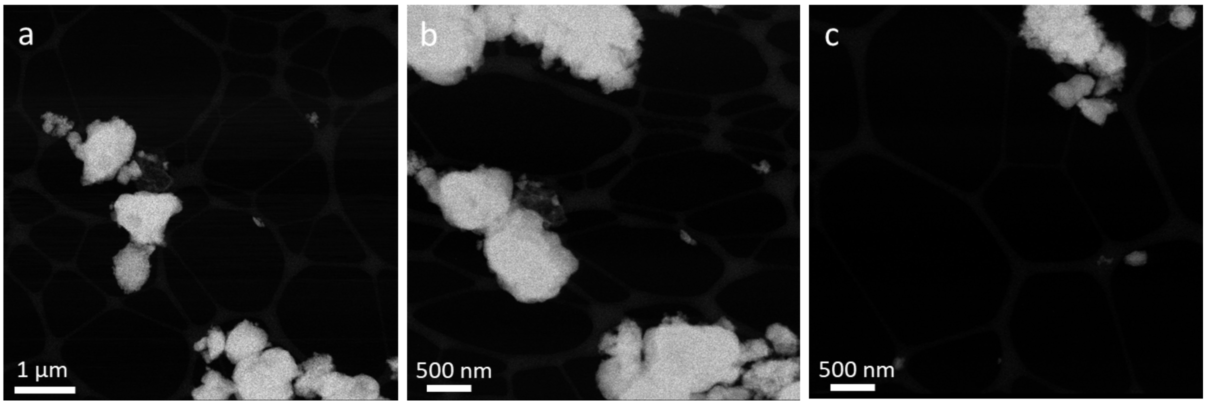

Figure S8. *ADF-STEM images of crystallites in the BCZT precursor mixture, quenched from 925 °C, with varying morphology, supported on a carbon-coated copper grid. Note that individual particles may contain multiple different phases and clear identification of oxycarbonate particles is only possible using electron diffraction, as shown in* ***Figure 8*** *of the main manuscript.*

References

1. Pasierb, P.; Komornicki, S.; Rokita, M.; Rękas, M., Structural properties of Li2CO3–BaCO3 system derived from IR and Raman spectroscopy. *Journal of Molecular Structure* **2001,** *596* (1), 151-156.

2. Jain, A.; Ong, S. P.; Hautier, G.; Chen, W.; Richards, W. D.; Dacek, S.; Cholia, S.; Gunter, D.; Skinner, D.; Ceder, G.; Persson, K. A., Commentary: The Materials Project: A materials genome approach to accelerating materials innovation. *APL Materials* **2013,** *1* (1), 011002.

3. Horton, M. K.; Huck, P.; Yang, R. X.; Munro, J. M.; Dwaraknath, S.; Ganose, A. M.; Kingsbury, R. S.; Wen, M.; Shen, J. X.; Mathis, T. S.; Kaplan, A. D.; Berket, K.; Riebesell, J.; George, J.; Rosen, A. S.; Spotte-Smith, E. W. C.; McDermott, M. J.; Cohen, O. A.; Dunn, A.; Kuner, M. C.; Rignanese, G.-M.; Petretto, G.; Waroquiers, D.; Griffin, S. M.; Neaton, J. B.; Chrzan, D. C.; Asta, M.; Hautier, G.; Cholia, S.; Ceder, G.; Ong, S. P.; Jain, A.; Persson, K. A., Accelerated data-driven materials science with the Materials Project. *Nature Materials* **2025,** *24* (10), 1522-1532.

4. Munro, J. M.; Latimer, K.; Horton, M. K.; Dwaraknath, S.; Persson, K. A., An improved symmetry-based approach to reciprocal space path selection in band structure calculations. *npj Computational Materials* **2020,** *6* (1), 112.

5. Materials Data on $BaCO_3$ by Materials Project. United States, 2020. DOI:10.17188/1208252.

6. Materials Data on BaO by Materials Project. United States, 2020. DOI: 10.17188/1189576.

7. Materials Data on $BaTiO_3$ by Materials Project. United States, 2020. DOI: 10.17188/1272706.
